# Divergence in poxvirus-encoded E3-like proteins can dictate poxvirus activation of cellular necroptosis

**DOI:** 10.1128/jvi.00114-26

**Published:** 2026-06-17

**Authors:** Junior A. Enow, Saige Munig, Mathew L. Sample, Gil Speyer, Raghavi C. Hundekar, Jacqueline Williams, James Bonner, Yize Li, Grant McFadden, Bertram Jacobs, Masmudur M. Rahman

**Affiliations:** 1 Molecular and Cellular Biology Program, Arizona State University 7864, Tempe, Arizona, USA; 2 School of Life Sciences, Arizona State University 7864, Tempe, Arizona, USA; 3 Center for Personalized Diagnostics, Biodesign Institute, Tempe, Arizona, USA; 4 Biodesign Center for Molecular Design and Biomimetics Biodesign Institute, Tempe, Arizona, USA; 5 Research Technology Office, Arizona State University 7864, Tempe, Arizona, USA; 6 ASU-Banner Neurodegenerative Disease Research Center Biodesign Institute, Tempe, Arizona, USA; Northwestern University Feinberg School of Medicine, Chicago, Illinois, USA

**Keywords:** necroptosis, poxvirus, myxoma virus, cell death, MYXV, dsRNA-binding proteins

## Abstract

Poxviruses encode a plethora of proteins adapted to diverse cellular responses against viruses. The poxvirus-encoded E3-like proteins are multifunctional, regulating diverse cellular antiviral responses. The canonical Vaccinia E3-like proteins have two domains: an N-terminal Z-form nucleic acid-binding domain (Zα-BD) and a C-terminal double-stranded RNA-binding domain (dsRNA-BD). Using protein sequence and structural homology modeling, we identified dsRNA-BD-fold-containing proteins in all the poxviruses except avipoxviruses, salmon poxvirus, and entomopoxviruses. Furthermore, we show that the acquisition of these proteins likely happened under three distinct events and can be classified into three categories: (i) the E3-like proteins with dsRNA-BD with the presence or absence of the N-terminal domain; (ii) unconventional/putative dsRNA-BD-fold-like proteins, present in macropoxvirus and molluscipoxvirus, and (iii) the dsRNA-BD-containing protein present in crocodile poxvirus. Members of leporipoxvirus, waddenpoxvirus, cetaceanpoxvirus, and selected members of orthopoxvirus contain E3-like proteins missing the N-terminal Zα-BD required for necroptosis inhibition. Using AlphaFold, we also show that the Zα-BD of chordopoxviruses, E3-like proteins, is structurally more variable than the dsRNA-binding domain. Compared to orthopoxviruses that inhibit necroptosis and contain an N-terminus Zα-BD, our results show that leporipoxviruses induce receptor-interacting protein kinase (RIPK) RIPK1- and RIPK3-mediated necroptosis in human and mouse necroptosis-competent cell lines. These data suggest that leporipoxviruses lack countermeasures against necroptosis, unlike orthopoxviruses, which encode multiple key regulators of necroptosis, possibly due to a lack of selective pressure in their lagomorph host species.

**IMPORTANCE:**

The evolutionary arms race between viruses and their hosts has led to the viral acquisition of genes that antagonize the host’s antiviral defenses. However, related viral and host proteins have evolved under positive, purifying, and neutral selection. Poxvirus-encoded E3-like proteins play a pivotal role in regulating the host’s cellular antiviral and apoptotic responses. The two domains of canonical E3-like proteins, an N-terminal Z-form nucleic acid-binding domain (Zα-BD) and a C-terminal double-stranded RNA-binding domain (dsRNA-BD), have evolved under varying host immune selection pressures. Here, we demonstrate that the dsRNA-BD is structurally conserved among E3 orthologs, whereas the Zα-BD, involved in regulating necroptosis, exhibits structural diversity and is absent in some poxviruses. Leporipoxviruses, which lack Zα-BD-containing proteins and evolved in species without functional necroptosis, do activate necroptosis in necroptotic-competent cells. Our study thus highlights that a lack of selective pressure from the host can shape viral countermeasures and viral divergence.

## INTRODUCTION

Poxviruses are unique among other DNA viruses in that they replicate exclusively in the cytoplasm of infected cells (1, 2). To successfully replicate, poxviruses encode many immune evasion proteins. The poxvirus-encoded vaccinia virus (VACV) E3-like family of proteins is one of the key immune evasion proteins. Vaccinia E3 protein has two functional domains: the N-terminal Z-form nucleic acid-binding domain (Zα-BD) and a C-terminal double-stranded RNA-binding domain (dsRNA-BD). The C-terminal dsRNA-BD is required to sequester dsRNA to avoid the activation of dsRNA-dependent antiviral proteins such as protein kinase R (PKR) (3–7). The N-terminal domain is required to inhibit virus-induced necroptosis (4, 8). Poxviral dsRNA-BD of E3 is present in all E3 orthologs, while the N-terminal Z-BD is missing in some E3 orthologs. Studies from different poxviruses suggest that the dsRNA-BD is essential for the *in vitro* replication and *in vivo* pathogenesis of poxviruses (4, 8–10). This is further confirmed by observations that the dsRNA-BD containing protein, NS1 from influenza virus, can rescue E3L-lacking VACV replication in cell culture (11). On the other hand, the N-terminal domain is dispensable for replication in most cell culture settings but required for VACV pathogenesis in mice (4, 6, 8, 10, 12–14).

Furthermore, the N-terminal domain is partially or entirely missing in some members of poxviruses (5, 7, 15). This N-terminal diversity among the E3 family of proteins suggests that these proteins might have adapted host-specific immune regulatory functions for *in vivo* pathogenesis in selected hosts. Additionally, the E3 protein family might be involved in regulating previously undescribed cellular pathways.

Necroptosis is a caspase-independent programmed cell death characterized by cell swelling, membrane rupture, and the release of pro-inflammatory molecules (16). Receptor-interacting protein kinase-3 and mixed-lineage kinase domain-like protein (MLKL) are the main drivers of necroptosis (16). Upon phosphorylation of RIPK3 by upstream stimuli, RIPK3 phosphorylates MLKL, and MLKL oligomerizes to the plasma membrane and terminates in cell lysis (17). Necroptosis can be activated via extracellular or intracellular pathways (18, 19). Activation of necroptosis by extracellular pathways is primarily mediated by death receptors and their ligands, like TNF-alpha and FasL ligand-receptor interactions; this cascade leads to the phosphorylation of RIPK1 and RIPK3 and the later phosphorylation of MLKL (16–18). Phosphorylated MLKL oligomerizes and localizes to the plasma membrane, leading to rupture (18). Intracellularly, Z-form nucleic acid accumulation can trigger the activation of Z-nucleic acid-binding protein 1 (ZBP-1) to activate RIPK3 and its associated downstream cascade. Since necroptosis-mediated activation of cell death is a cellular defense mechanism against viruses, some virus-encoded proteins can regulate necroptosis, such as the N-terminal domain of VACV E3 protein (4, 8). Poxviruses also encode additional proteins, such as vIRD (viral inducer of RIPK3 degradation) and vMLKL (viral MLKL), that regulate cellular necroptosis at multiple points in the pathway (20, 21).

We previously demonstrated that specific mammalian lineages, including leporids, cetaceans, and certain rodents, harbor inactivating mutations in either RIPK3 or MLKL, rendering necroptosis non-functional in these species (3). Simultaneously, we demonstrated a correlation between the lack of the N-terminal domain of E3-like proteins required for necroptosis inhibition among leporipoxviruses (3). However, members of leporipoxviruses were not tested for their ability to stimulate necroptosis in necroptosis-competent cells.

Here, we identify two unique dsRNA-fold-containing proteins within Chordopoxviruses, in addition to the traditional E3 protein. We show that the Zα-BDs of Chordopoxvirus E3 proteins are structurally more variable than the dsRNA-BD. The AlphaFold structure of E3 proteins from poxviruses lacking the Zα-BDs shows unique modifications at the N-terminus region.

Compared to VACV and cowpox virus (CPXV), which have been shown to inhibit necroptosis, members of leporipoxviruses activate necroptosis in necroptosis-competent human and mouse cells (4, 20). Furthermore, MYXV (a model leporipoxvirus) infection activates RIPK1- and RIPK3-mediated necroptosis in these cells, suggesting that leporipoxviruses lack a countermeasure against cellular necroptosis.

## RESULTS

### E3-like proteins encoded by the members of Chordopoxviruses can be classified into three families based on the origin of the structurally conserved C-terminus dsRNA-BD

Poxvirus E3-like proteins are critical for virus replication and pathogenesis among poxviruses (6, 22, 23). To understand the diversity of E3-like proteins across poxviruses, we performed a sequence homology search using the Basic Local Alignment Search Tool (BLAST) protein-protein BLAST and Domain-Enhanced Lookup Time Accelerated (DELTA)-BLAST using VACV E3 protein as a query against the poxvirus proteome on the National Center for Biotechnology Information (NCBI) database (A detailed description of our BLAST process is provided in the Materials and Methods section).

**Fig 1:**
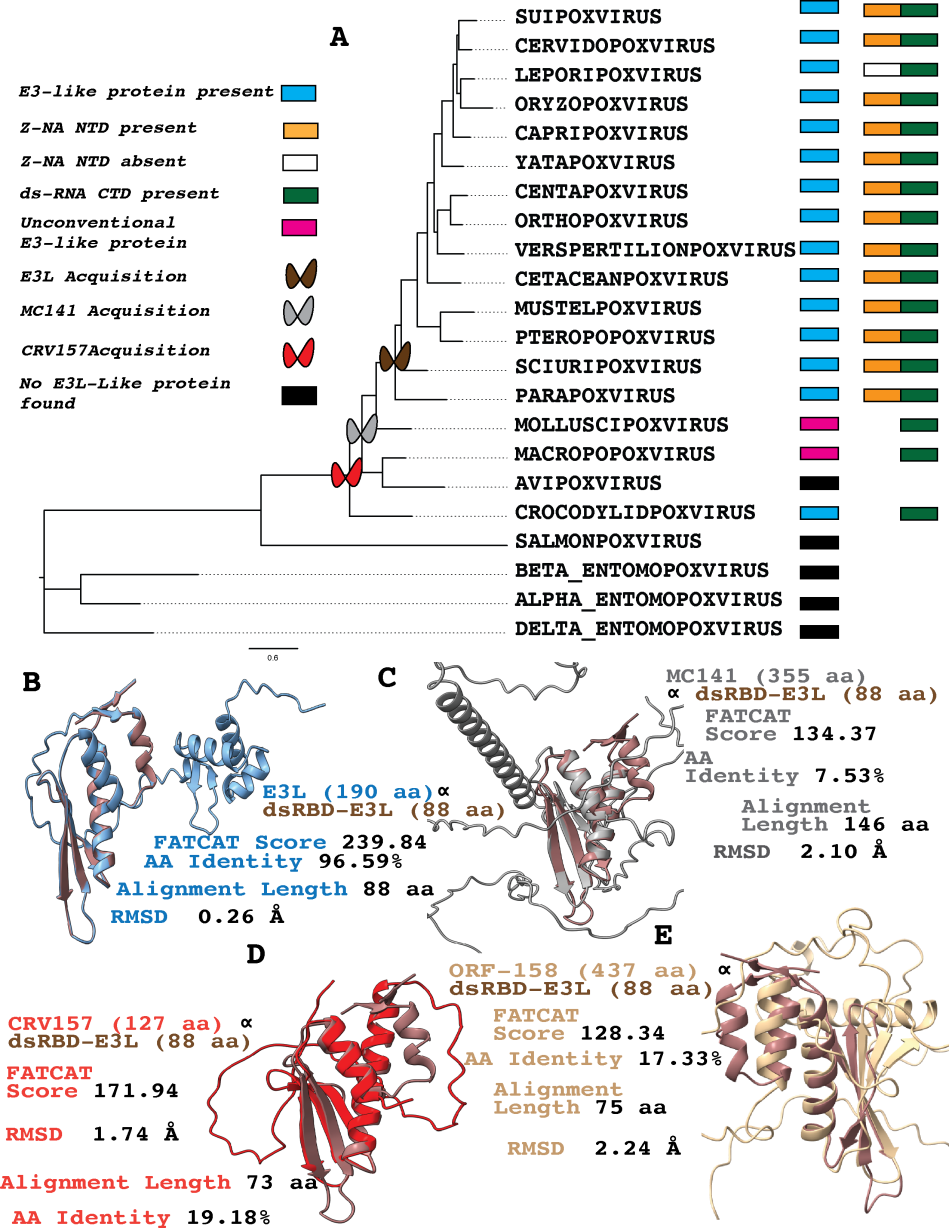
Diversity of dsRNA-BD fold-containing proteins within Poxviruses. (**A**) Phylogenetic tree of all poxvirus families showing the acquisition of dsRNA-BD proteins. Structural homology showing FATCAT score, amino acid identity, alignment length, and root mean square deviation (RMSD) value comparing the dsRNA-BD of VACV to (**B**) VACV E3, (**C**) Molluscipoxvirus MC141, (**D**) Crocodilepoxvirus CRV157, and (**E**) Macropoxvirus ORF-158.

Our amino acid (aa) sequence screen revealed that avipoxviruses, salmonpoxvirus, and entomopoxviruses did not contain an E3-like/dsRNA-fold containing protein (Fig. 1A), suggesting that a different viral protein might be involved in viral nucleic acid regulation in these viruses or that the mechanism of dsRNA regulation differs from the canonical model.

Due to the lack of sequence similarity among proteins that share similar structural domains, we built 11,001 reference poxvirus structures from different poxvirus genera using AlphaFold 2.

**Fig 2:**
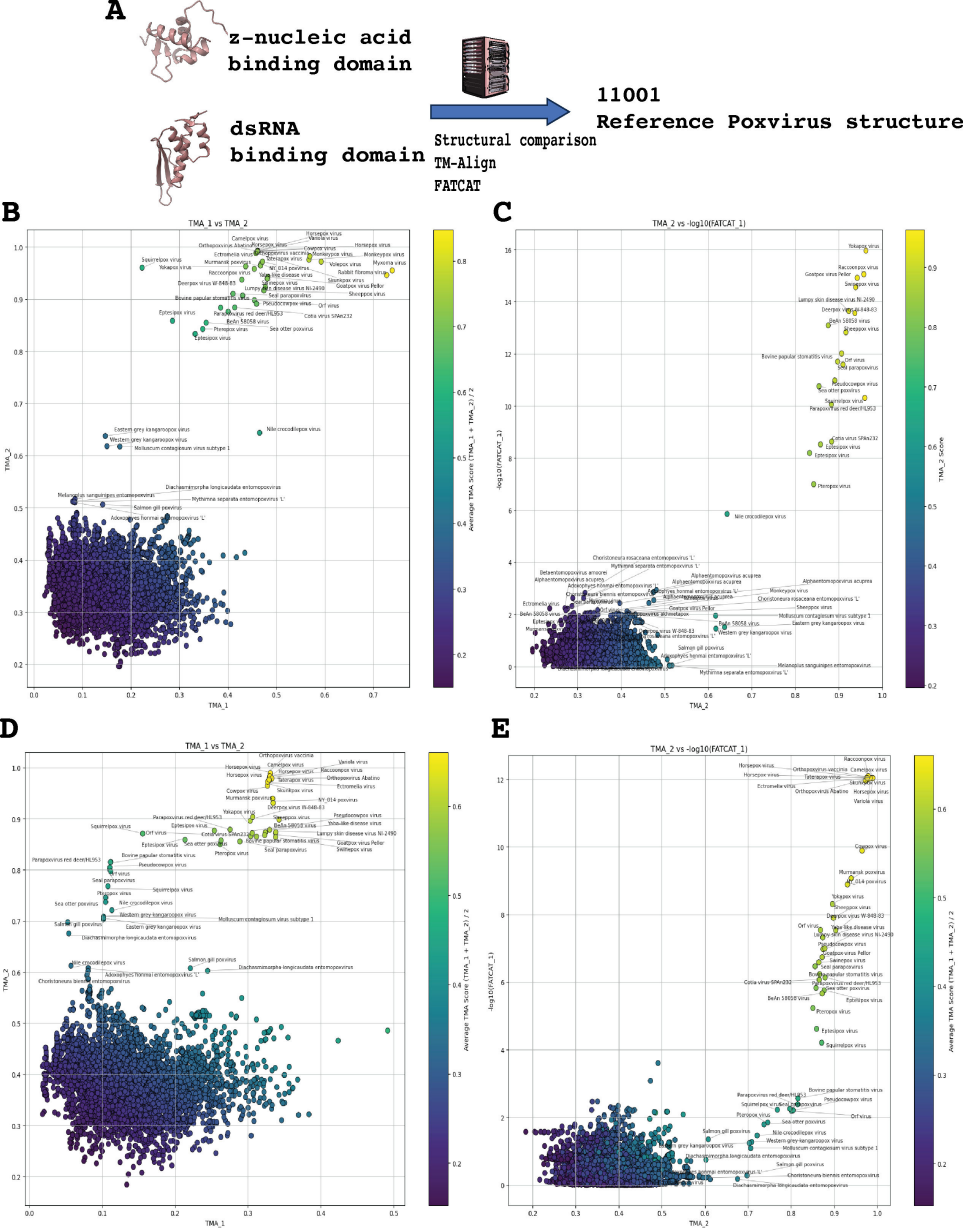
Structural homology screen using the N- and C-terminus of VACV E3 proteins as query against reference poxvirus protein structures. (**A**) Schematic illustrating how the structural homology screen was performed. (**B**) A plot of TM-align 2 values against TM-align 1 values, and (**C**) showing the negative log of the FATCAT *P*-value against TM-align 2 value for the E3 protein C-terminus domain as query, against the poxvirus structural repository, while (**D**) and (**E**) use the N-terminus domain of E3 as query.

To validate our initial sequence alignment search result, we performed structural homology comparisons as implemented by the FATCAT (Flexible Structure Alignment by Chaining Aligned Fragments Pairs with Twist) and TM-align programs, using the dsRNA-BD and Zα-BD domains of the VACV E3 as a query protein against our repository of reference poxvirus structures (Fig. 2A) (24–26). Contrary to what was observed in our sequence alignment screen, entomopoxvirus and salmonpoxvirus contained proteins that shared a significant structural similarity to the dsRNA-BD of VACV-E3, leaving avipoxvirus as the only member of the poxvirus family without a significant dsRNA-BD fold-containing protein (Fig. 2B and C). It is worth noting that the newly identified dsRNA-BD fold structural proteins discovered within the entomopoxvirus and salmonpoxvirus families differ in the secondary structure configurations from the canonical E3 dsRNA-BD protein. Hence, we decided that their analysis would be the subject of a subsequent inquiry.

**Fig 3:**
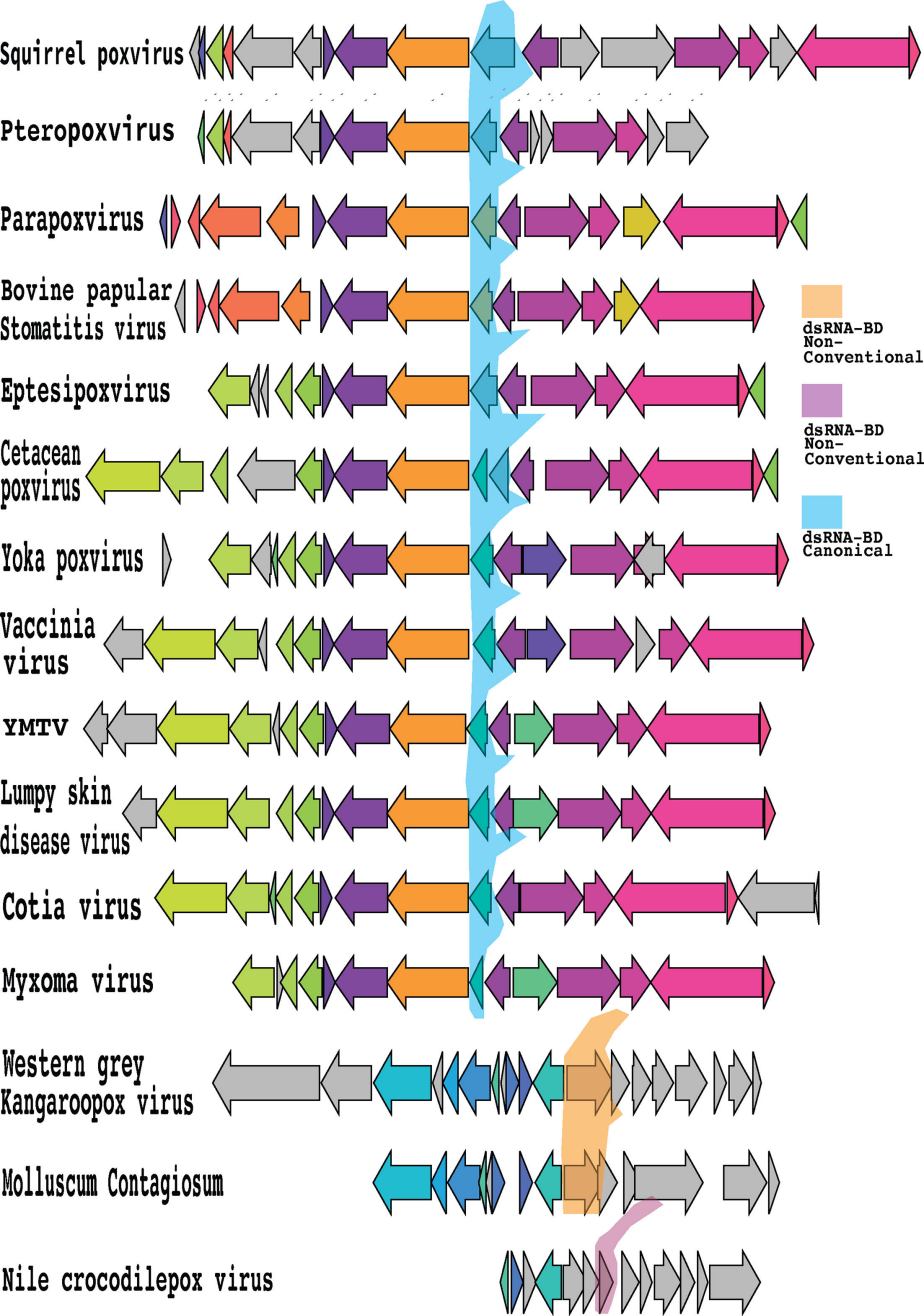
Origin of poxvirus dsRNA-BD-containing proteins. Synteny analysis showing the relatedness of the genes surrounding poxvirus dsRNA-BD-containing proteins. Blue highlighted region indicating the canonical poxviral dsRNA-binding proteins, while the orange and pink highlights represent non-conventional poxviral dsRNA-fold-containing proteins.

**Fig 4:**
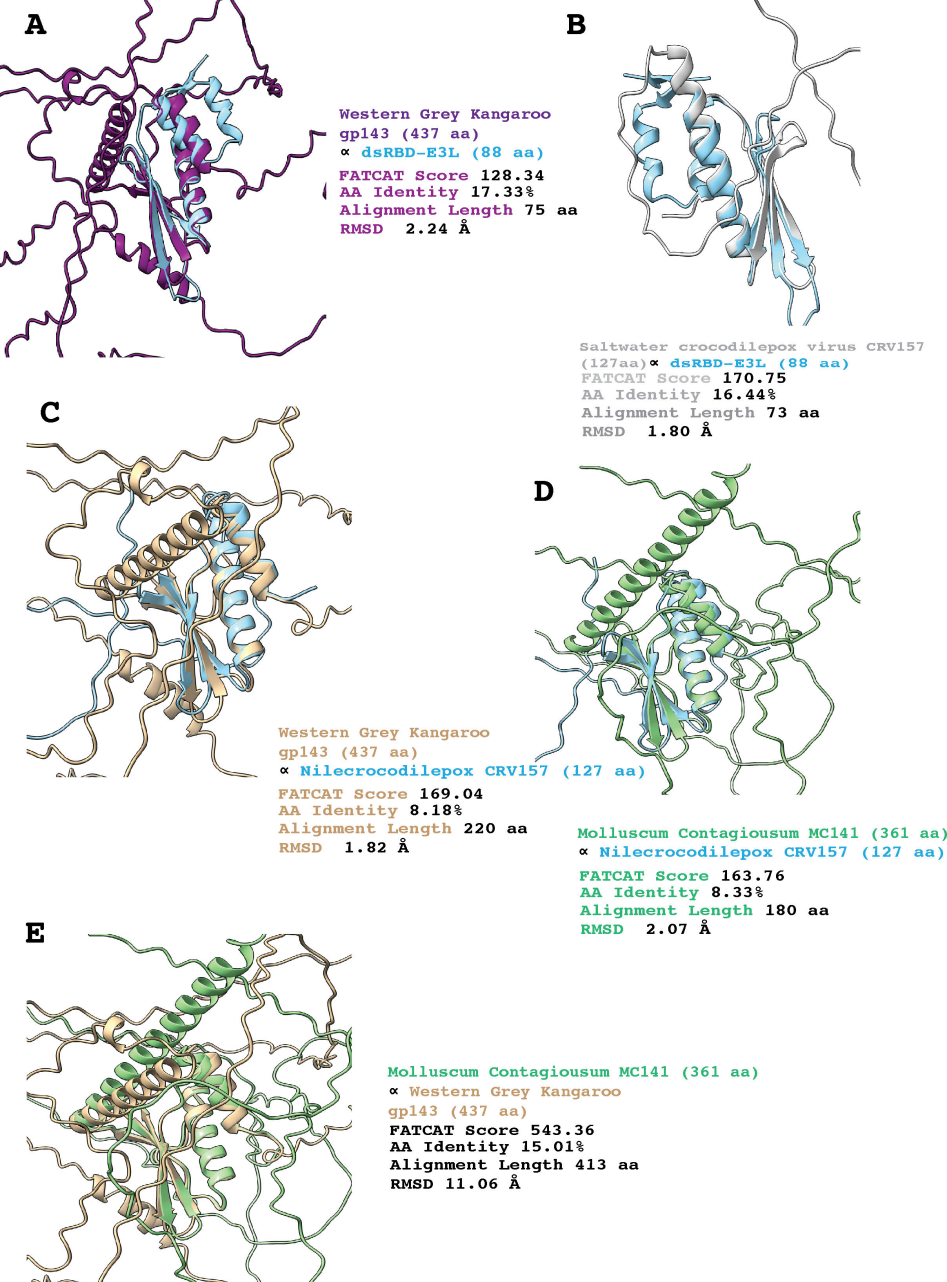
Structural comparison of dsRNA-BD-fold containing proteins within poxviruses. Structural homology showing FATCAT score, amino acid identity, alignment length, and RMSD value comparing the dsRNA-BD of VACV E3 to (**A**) Western Grey Kangaroo poxvirus gp143, (**B**) saltwater crocodile pox virus CRV157. FATCAT comparison between gp143 and CRV157 (**C**), MC141 and CRV157 (**D**), and MC141 compared to gp143 (**E**).

Both our structural homology and aa screens identified unique dsRNA-BD-fold-containing protein structures belonging to macropoxvirus and molluscipoxvirus, which differ from the canonical E3-like proteins found in most poxviruses (Fig. 1B, C and E). Given the lack of sequence and structural similarity among all the E3-like proteins in our screen, we asked whether poxviruses share a common E3 ancestor. Interestingly, our synteny analysis revealed that poxvirus dsRNA-BD-fold-containing proteins had three distinct acquisition events (Fig. 1A and Fig. 3). Members of all Chordopoxviruses except molluscipoxvirus, macropoxvirus, avipoxvirus, crocodile poxvirus, and salmonpoxvirus share the same ancestral E3-like protein as VACV (Orthopoxviruses), with an E3L gene duplication in the cetacean poxvirus family (Fig. 1A and Fig. 3). Molluscipoxvirus and macropoxvirus share a unique dsRNA-BD-fold-containing protein (MC141 and ORF158) present in no other poxvirus family (Fig. 1A, C and E, Fig. 4D and E). Crocodile poxvirus contains a dsRNA-BD-containing protein (CRV157) like E3 but with a different origin (Fig. 1A and D and Fig. 3) (27).

We performed FATCAT structural comparison using the dsRNA-BD of VACV E3 as our standard model and compared it with the dsRNA-BD-containing proteins discovered in our screen (the FATCAT is the best of all alignment possibilities between a query and target structure, with a *P*-value score dictating the significance of the two structures being compared with unrelated structures. Higher FATCAT scores suggest better alignments). Molluscipoxvirus MC141 had a significant FATCAT score of 134.37, an root mean square deviation (RMSD) of 2.10 Å, and an aa identity of 7.53%, suggesting a shared dsRNA-BD homology with VACV dsRNA-BD (Fig. 1C). Crocodile poxvirus CRV157 had a significant FATCAT score of 171.94, an aa identity of 19.18%, and an RMSD score of 1.74 Å (Fig. 1D). Macropoxvirus ORF-158 was significantly similar to VACV-dsRNA-BD (FATCAT Score 128.34, 17% aa identity, and 2.24 Å RMSD) (Fig. 1E). Western grey kangaroo poxvirus gp143 and saltwater crocodile poxvirus CRV157 were also significantly related to VACV-dsRNA-BD (Fig. 4A and B).

Additionally, we focused on the residues that have previously been shown to be involved in dsRNA binding (28). We aligned the sequences of 11 poxviruses (Wadden Sea poxvirus [WSPV], Western grey kangaroo poxvirus [WKPV], volepoxvirus [VPXV], vaccinia virus [VACV], saltwater crocodile poxvirus [SCPV], Nile crocodile poxvirus [NCPV], monkeypox virus [MPV], molluscum contagiosum virus [MCV], cetacean poxvirus [CEPV], cowpoxvirus [CPV], and myxoma virus [MYXV]) dsRNA-fold-containing proteins from our screen using Multiple Sequence Comparison by Log-Expectation (MUSCLE). Western grey kangaroo poxvirus gp143 dsRNA-fold contained 2 of the six residues critical for dsRNA binding (Phe-148 and Lys-171, residues 180 and 203, respectively, on the consensus sequence map), while molluscipoxvirus MC141 had three of the six dsRNA-binding amino acids (Phe-135, Phe-148, and Lys-171, residues 167, 180, and 203, respectively, on the consensus sequence map) (Fig. S1A). Clustal Omega alignment using the previously described 12 sequences showed that gp143 and MC141 contained the glu-124 residue (residue 200 on the consensus sequence map) required for dsRNA binding (Fig. S1B). In addition, we show that the residues that match the dsRNA-binding residues from the E3 protein share a similar structural location on the predicted protein structure of gp143 and MC141 (Fig. S2).

### Recurrent loss of the Z-form nucleic acid-binding domain (Zα-BD) of the E3-like proteins required for intracellular necroptosis inhibition

**Fig 5:**
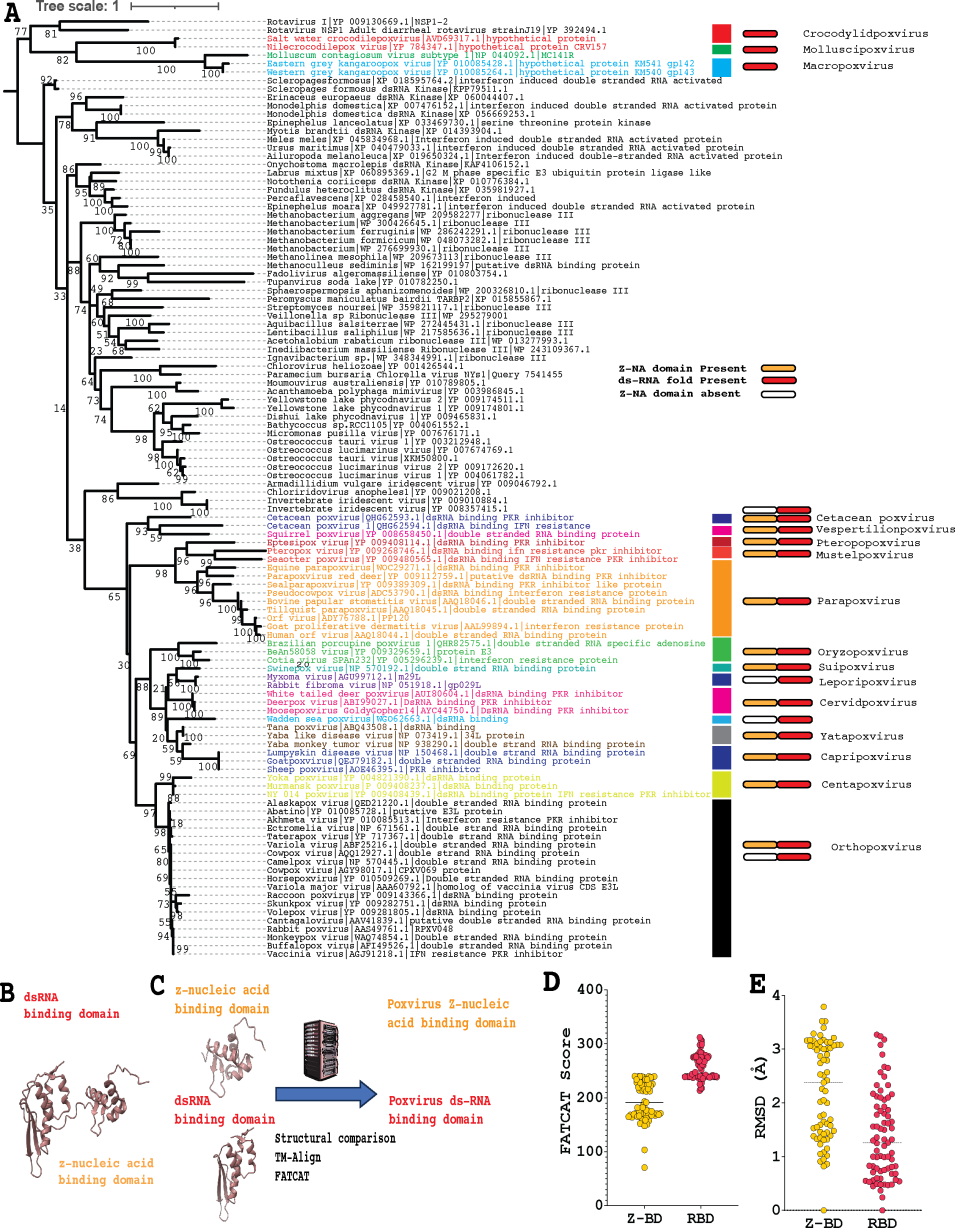
Recurrent loss of Zα-BD of E3-like proteins. (**A**) Phylogenetic tree of poxvirus dsRNA-BD containing proteins from diverse virus and host species. (**B**) AlphaFold generated structure of VACV E3 protein depicting the dsRNA-BD (RBD) (red) and Zα-BD (Z-BD) (yellow). (**C**) Figure depicting FATCAT and TM-Align structural comparisons of VACV Zα-BD and dsRNA-BD to poxvirus proteins containing homologs to these domains. (**D**) FATCAT score and (**E**) RMSD value of all poxvirus Zα-BD and dsRNA-BD compared to VACV E3 standard.

Our previous work demonstrated a correlation between the loss of RIPK3 and MLKL among certain mammalian species and the corresponding lack of a Zα-BD of the accompanying infecting poxvirus (3). We performed phylogenetic analysis using the poxvirus E3-like proteins from our initial screen to gain an understanding of how the loss of the Zα-BD varies across different species of poxviruses. Phylogenetic tree construction of E3 proteins showed a pattern of loss of the Zα-BD at different poxvirus genera; for example, within the orthopoxvirus genus, cowpoxvirus has an N-terminus Zα-BD, while volepoxvirus lacks an N-terminus of E3. This suggests that the losses of the E3 N-terminus regions of Chordopoxviruses are independent events occurring in different genera (Fig. 5A).

Using the Zα-BD of E3 as a query, we performed a structural homology screen against the N-terminus of E3-related proteins using our poxvirus protein structure repository, and we excluded structures with fewer than five secondary structural elements. Our screen revealed the presence of Zα-BD-related domains in multiple poxvirus genera (Fig. 2D and E). Surprisingly, we observed that the N-terminus Zα-BD structures of several E3-related proteins in our screen contained additional secondary structures not present in the N-terminal Zα-BD of VACV-E3 (the standard Z-NA model) (most notably the E3-related proteins from squirrelpox virus, pteropox virus, sea otter poxvirus, and yokapox virus). To understand the extent of structural diversity in the N-terminus domain of the poxvirus E3 protein, we performed a structural homology comparison using FATCAT and TM-align. The E3 proteins were broken into the N-terminus Zα-BD and C-terminus dsRNA-BD. The N- and C-termini of E3 were used as queries and compared with all poxviruses that contain N- and C-termini, respectively, Zα-BD and dsRNA-BD (Fig. 5B and C). Our results show a lower FATCAT score and a higher RMSD value for the N-terminus domain compared to the C-terminus, indicating lower structural similarity at the Zα-BD (Fig. 5D and E). The lower FATCAT scores at the N-terminus domain of E3 suggest modifications to the Zα-BD that might alter its function.

**Fig 6:**
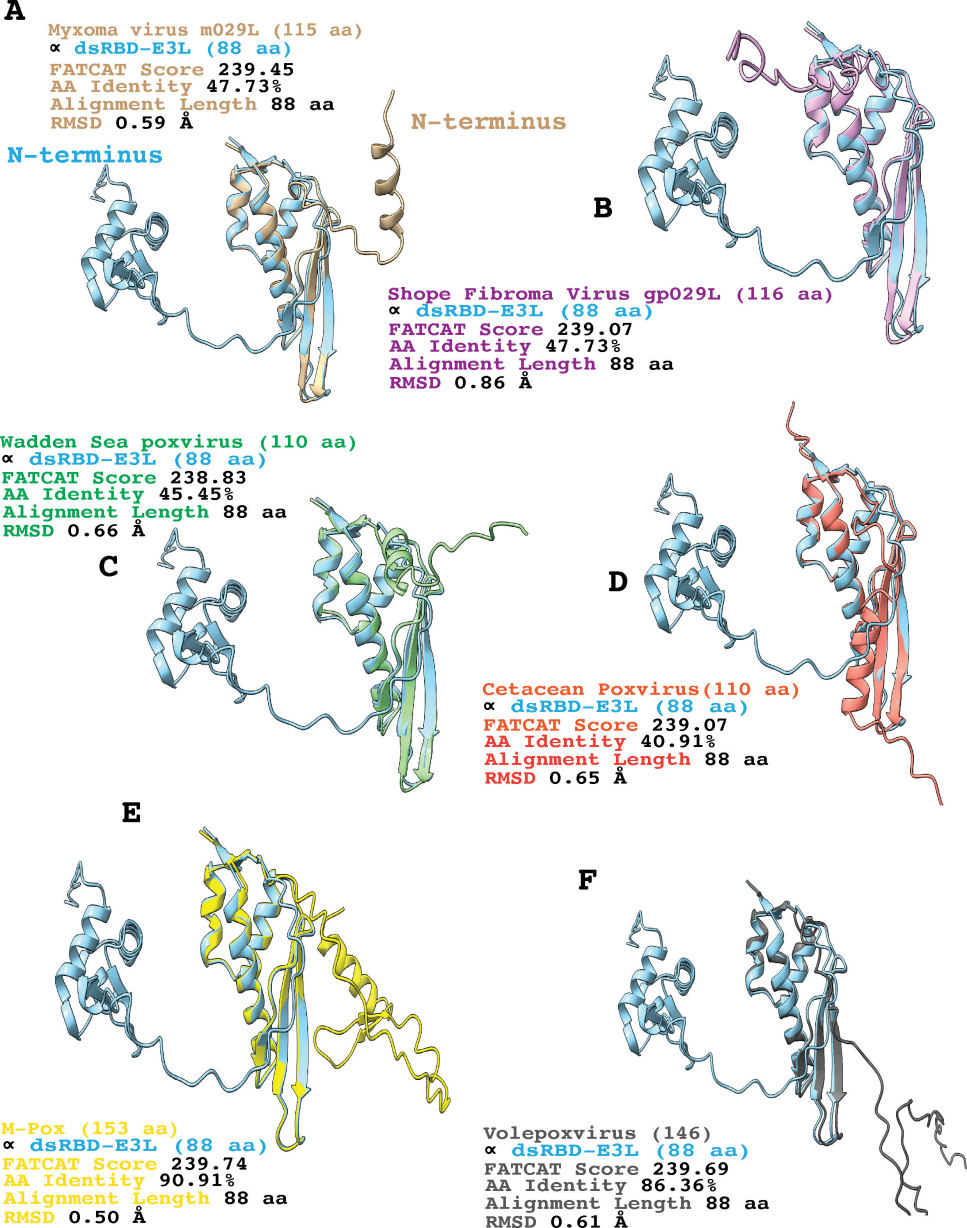
Recurrent loss of the Zα-BD among Chordopoxviruses. Structural homology showing FATCAT score, amino acid identity, alignment length, and RMSD value comparing the dsRNA-BD of VACV to (**A**) Myxoma virus M029, (**B**) Shope Fibroma virus gp029, (**C**) Wadden Sea poxvirus E3-like proteins, (**D**) Cetacean poxvirus E3-like proteins, (**E**) Mpox virus E3-like proteins, and (**F**) Volepoxvirus E3-like proteins.

Leporipoxvirus family members (MYXV and SFV) and Wadden Sea poxvirus lack an N-terminus Zα-BD domain, although structural comparison with VACV dsRNA-BD shows a significant structural similarity (Fig. 6A through C). The structural model of MYXV E3-like protein M029 forms an alpha helix at the N-terminus that is not present in SFV gp029, its close cousin (Fig. 6A and B). Wadden Sea poxvirus E3-like protein also lacks an N-terminus region (Fig. 6C). Cetaceanpoxvirus contains two copies of the E3-like protein, one harboring a complete Zα-BD and the other missing the N-terminus region (Fig. 6D). On the other hand, although most members of orthopoxviruses possess an intact Zα-BD, some members are missing part (Mpox) or the entire N-terminal region of the Zα-BD (volepox virus) (Fig. 6E and F). Together, the data suggest diversity in the N-termini of E3 protein orthologs within poxviruses.

### Leporipoxviruses activate necroptosis in necroptosis-competent human cells

**Fig 7:**
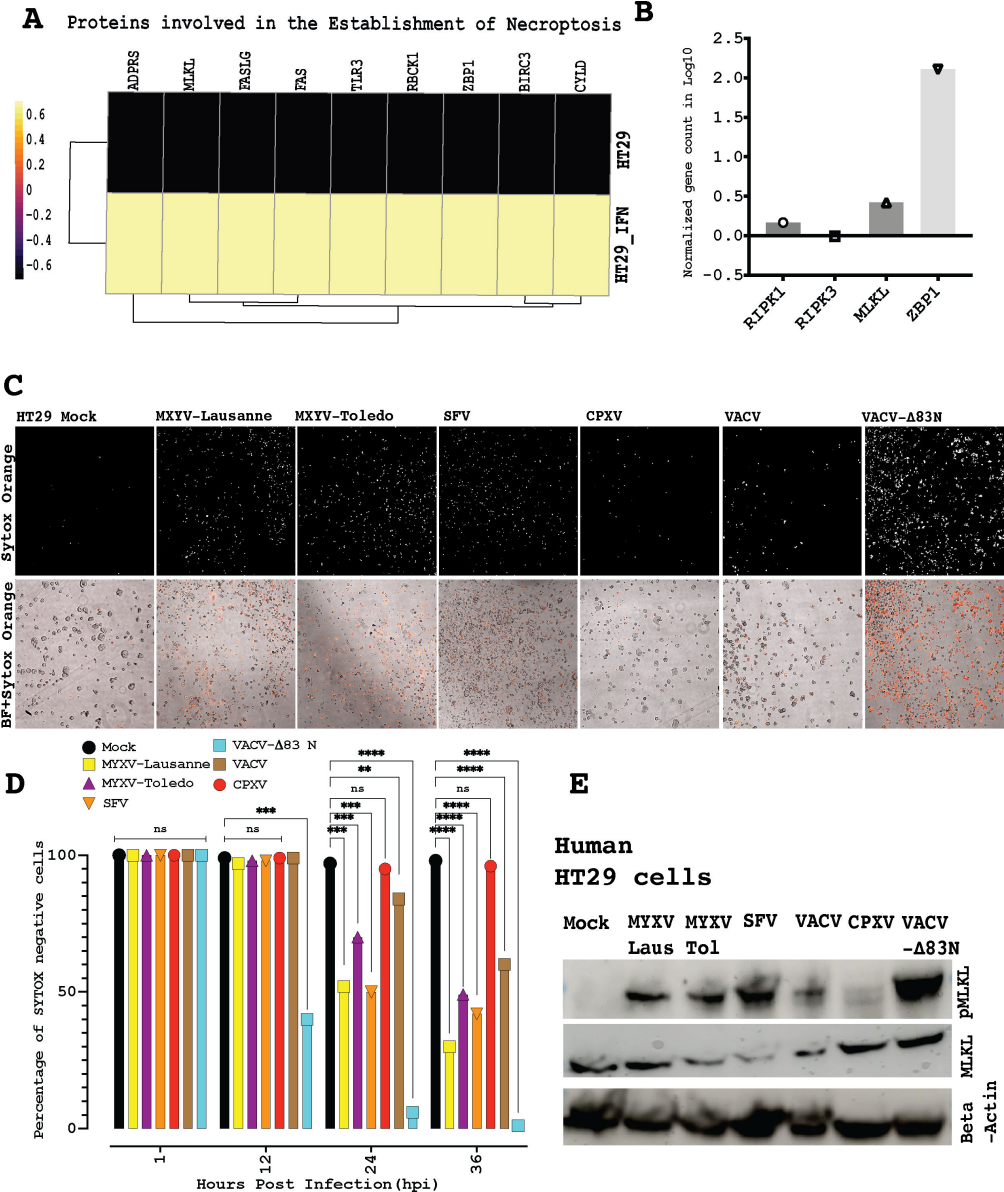
Leporipoxviruses stimulate necroptosis in necroptosis-competent human HT29 cells. (**A**) Heatmap depicting differentially expressed genes involved in establishing necroptosis after treatment with IFN and (**B**) Log fold change in RIPK1, RIPK3, MLKL, and ZBP-1 in human HT29 cells. (**C**) Fluorescence images of interferon-primed HT29 cells, either uninfected (mock) or infected with MYXV-Lausanne, MYXV-Toledo, SFV, CPXV, VACV, and VACV-83N. Samples were stained with Sytox Orange dye and imaged 24 h.p.i. (**D**) Viability of mock and virally infected HT29 cell samples collected at 1, 12, 24, and 36 h after virus infection. (**E**) Western blot probing for phosphorylated MLKL, MLKL, and beta-actin levels in infected and uninfected samples.

**Fig 8:**
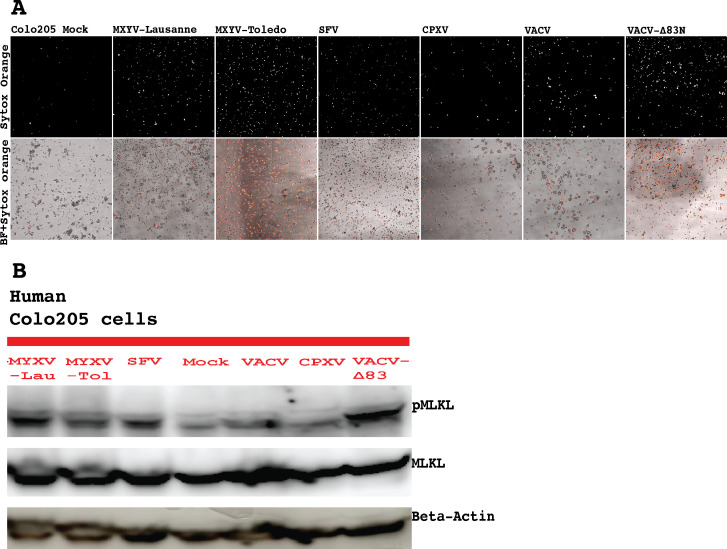
Leporipoxviruses activate necroptosis in necroptosis-competent human Colo205 cells. (**A**) Fluorescence images of interferon-primed Colo205 cells, either uninfected (mock) or infected with MYXV-Lausanne, MYXV-Toledo, SFV, CPXV, VACV, and VACV-83N. Samples were stained with Sytox Orange dye and imaged at 24 h.p.i. (**B**) Western blot probing for pMLKL, MLKL, and beta-actin levels in infected and uninfected IFN-primed Colo205 cells.

Compared to orthopoxviruses, such as VACV and CPXV, which have previously been shown to inhibit necroptosis and contain an E3 protein with an N-terminus Zα-BD, it is not known whether different members of leporipoxviruses induce necroptosis (3, 8, 20). To investigate whether leporipoxviruses activate necroptosis, we utilized two human cell lines, HT29 and Colo205, that are competent for necroptosis. We sequenced the transcriptome of HT29 cells in the presence and absence of human interferon beta (IFN-) to obtain an expression profile of the genes involved in necroptosis. Our data showed that MLKL and ZBP1, the effector protein of necroptosis and host sensor of Z-nucleic acid, respectively, were IFN-inducible genes (Fig. 7A and B). Human HT29 cells were primed with human IFN- for 24 h and infected with representative members of leporipoxviruses (MYXV-Lau, MYXV-Tol, and SFV) and orthopoxviruses known to inhibit necroptosis (VACV and CPXV). An hour before infection, cells were treated with a pan-caspase inhibitor, z-VAD-FMK, and infected with the stated poxviruses. At 1, 12, 24, and 36 h post-infection, cells were treated with sytox orange to differentiate the necroptosis-positive and negative cells and imaged. At 24 h post-infection, leporipoxviruses (MYXV-Lau, MYXV-Tol, and SFV) had more SYTOX-positive (necroptosis-positive) cells than CPXV and VACV (orthopoxvirus) (Fig. 7C). However, VACV-Δ83N (VACV lacking the N-terminus Zα-BD), the positive control for virus-induced necroptosis, had the highest amount of SYTOX-positive cells (Fig. 7C). Quantification of cells at 1, 12, 24, and 36 h post-infection with MYXV-Lau, MYXV-Tol, and SFV showed less than 50% necroptosis-negative cells at 36 hpi. In contrast, wild-type CPXV had more than 98% necroptosis-negative cells at 36 hpi, values comparable to those of untreated cells. However, wild-type VACV showed a marked 35% decrease in necroptosis-negative cells compared to CPXV, while 99% of the cells were necroptosis-positive in the VACV-83N infection condition at 36 h (Fig. 7C and D). We then monitored MLKL phosphorylation levels to confirm necroptosis. HT29 cells were collected at 24 hpi in the presence of phosphatase and protease inhibitors and subjected to western blot analysis. The results showed that MYXV-Lau, MYXV-Tol, SFV, and VACV-83N infections led to the accumulation of phosphorylated MLKL (pMLKL); infection with WT-VACV resulted in a trace amount of pMLKL accumulation, whereas infection with CPXV produced little to no detectable MLKL phosphorylation (Fig. 7E). We repeated the same experiment in human Colo205 cells and observed a similar pattern of cell viability and MLKL phosphorylation (Fig. 8A and B). Taken together, the data demonstrate that leporipoxviruses activate necroptosis in human HT29 and Colo205 cells, suggesting that leporid-infecting poxviruses lack countermeasures to necroptosis.

### MYXV activates both RIPK1- and RIPK3-mediated necroptosis in human cells

**Fig 9:**
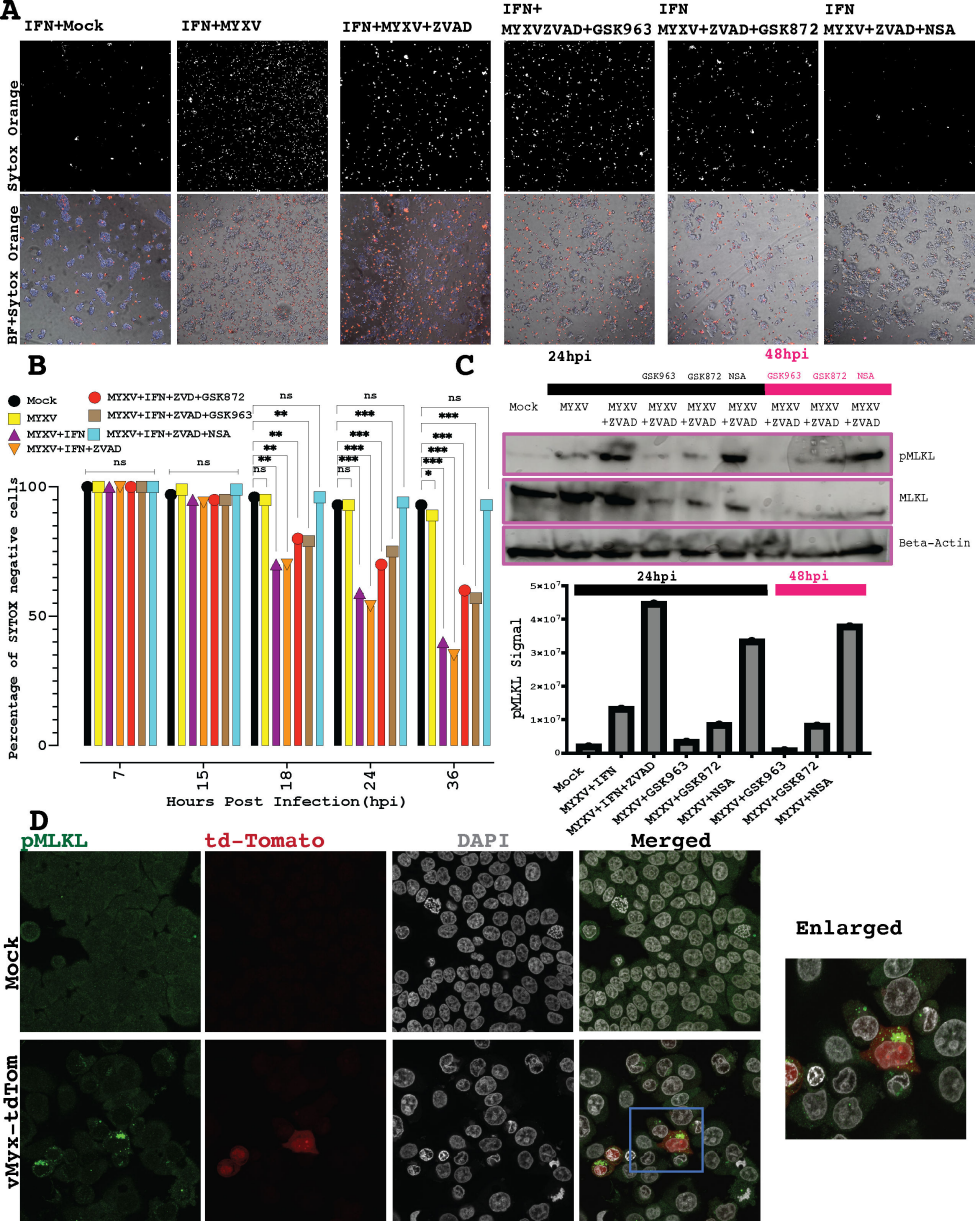
MYXV activates both RIPK1- and RIPK3-mediated necroptosis in necroptosis-competent human cells. (**A**) Fluorescence images of IFN-primed HT29 cells: mock uninfected, MYXV-infected, MYXV-infected, and treated with ZVAD, MYXV infection in the presence of ZVAD and GSK963 or GSK872 or NSA. Samples were imaged 24 h.p.i in the presence of Sytox Orange. (**B**) Experiment A was repeated to measure viability by collecting samples at 7, 15, 18, 24, and 36 h after MYXV infection. (**C**) Western blot of IFN-primed HT29 cells with the same treatments as in A, and probing for human pMLKL, MLKL, and beta-actin levels, harvested at 24 and 48 h.p.i., with pMLKL signal intensity measured using Image Studio (bottom panel). (**D**) Confocal images of pMLKL in mock and myxoma virus-infected IFN-primed HT29 cells, fixed at 24 h after virus infection.

Since leporipoxviruses have evolved in species that are missing core executors of the necroptotic pathway, we tested how key proteins of the necroptotic pathway would affect the activation of necroptosis in the presence of MYXV-Lau, a model leporipoxvirus. We used known inhibitors of RIPK3, RIPK1, and MLKL in human HT29 cells to assess their effects on MYXV-mediated activation of necroptosis. We pretreated IFN-primed human HT29 cells with inhibitors of RIPK1 (GSK’963), RIPK3 (GSK’872), and MLKL (Necrosulfonamide [NSA]) in the presence of z-VAD FMK, or cells left untreated for 1 h. Next, the cells were infected with MYXV-Lau, and Sytox viability fluorescence images were taken at different time points. MYXV infection led to a greater than 60% decrease in necroptosis-negative cells at 36 h post-infection (Fig. 9A and B). However, inhibition of RIPK1 or RIPK3 increased cell viability and the number of necroptosis-negative cells compared to infection with the virus alone at 36 h. Surprisingly, inhibition of MLKL via NSA restored MYXV-infected necroptosis-negative cells to about 98%, 36 h after infection (Fig. 9A and B). MYXV infection stimulated the accumulation of phosphorylated MLKL, even in the absence of z-VAD-FMK treatment, suggesting that caspase inhibition is not critical for MYXV-activated necroptosis (Fig. 9C). However, Z-VAD inhibition increased the amount of accumulated pMLKL (Fig. 9C), indicating that other death pathways are activated by MYXV infection.

The inhibition of RIPK1 and RIPK3 reduced pMLKL accumulation, while treatment with NSA accumulated pMLKL, although it significantly reduced necroptosis to greater than 98%. This is consistent with previous reports showing that MLKL is phosphorylated in the presence of NSA, but the effector function is inhibited (Fig. 9A through C) (29, 30). Immunofluorescence staining of pMLKL shows it is localized to the cytoplasmic compartment and forms punctate structures 24 h post-MYXV infection (Fig. 9D), consistent with its oligomerization before effector function. In summary, our data demonstrate that in human necroptosis-competent HT29 cells, MYXV activates RIPK1 and RIPK3 necroptosis.

### RIPK1 and RIPK3 blockage rescues MYXV-activated necroptosis in necroptosis-competent mouse cells

**Fig 10:**
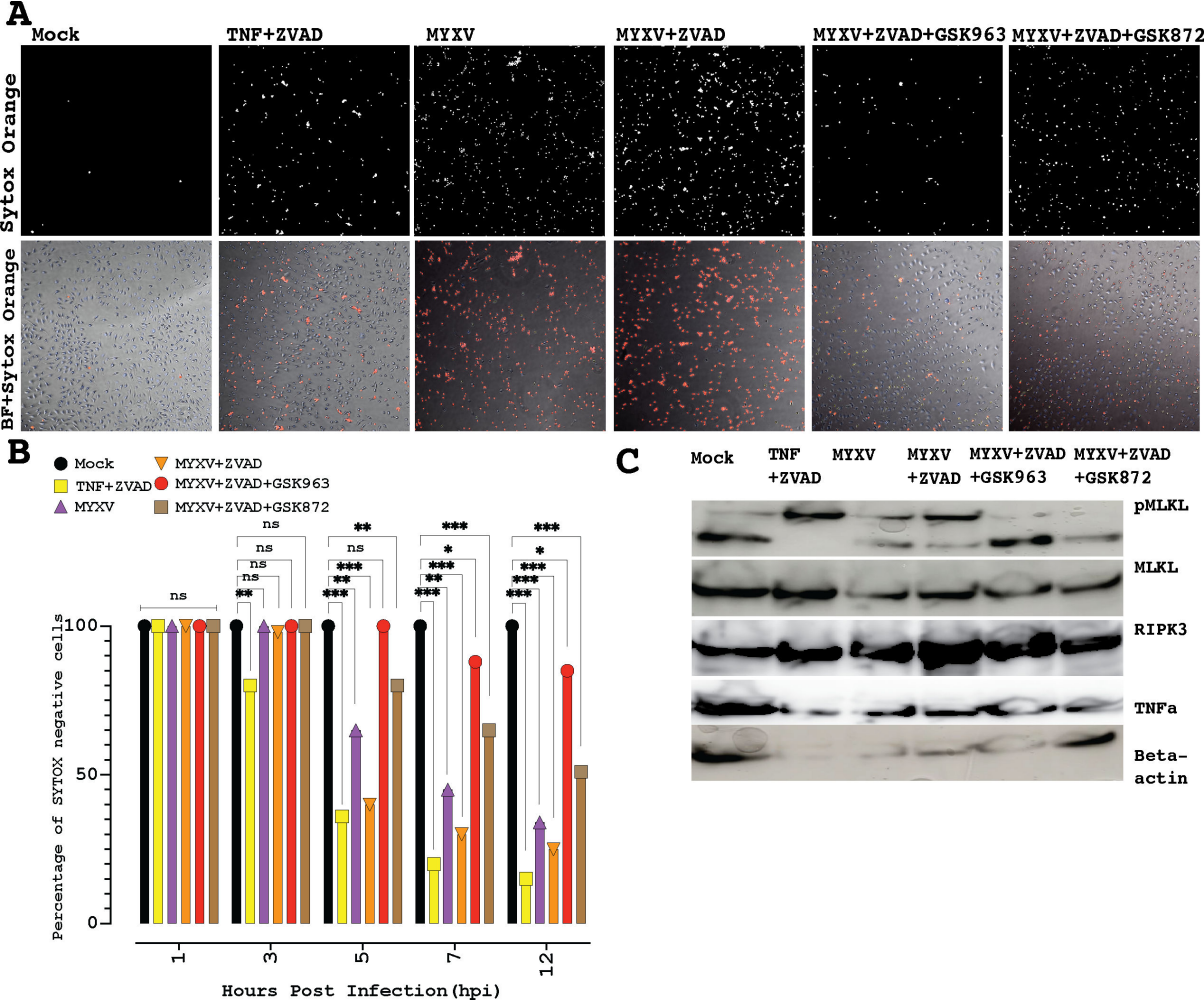
Inhibition of necroptosis rescues MYXV replication in mouse L929 cells. (**A**) Fluorescence images of IFN-primed L929 cells with the following treatments: untreated (Mock), ZVAD and TNF, MYXV, MYXV + ZVAD, MYXV + ZVAD + GSK963, and MYXV + ZVAD + GSK872. At 12 h post-treatment, cells were treated with Sytox Orange dye and images were captured. (**B**) In a separate experiment, as described in A, cell viability was calculated at 1, 3, 5, 7, and 12 h after infection, by measuring cells that were SYTOX-positive, related to the total cell number. (**C**) Western blot analysis for detecting the presence of pMLKL, MLKL, RIPK3, TNF, and beta-actin.

Next, we tested whether MYXV infection can activate necroptosis in mouse cells and whether the components of necroptosis activation function like those of human cells. In this case, we used necroptosis-competent mouse L929 cells (4). Mouse IFN-primed L929 cells were either left untreated or treated with z-VAD in the presence of GSK963 and GSK872. As previously observed in human cells, MYXV-activated necroptosis did not depend on caspase inhibition (Fig. 10A and C), suggesting that MYXV-encoded caspase inhibitors are functional in mouse-derived cells. At 12 h post-infection, with or without interferon treatments, the viability of MYXV-infected L929 cells was significantly reduced to only 25% (Fig. 10B). RIPK1 inhibition via GSK’963 led to a significant increase in cell viability (more than 80% necroptosis-negative cells) compared to MYXV infection alone (Fig. 10B). Additionally, inhibition of RIPK3 with GSK’872 increased cell viability (approximately 50% necroptosis-negative cells) compared with virus-only conditions (Fig. 10B). We confirmed that the cell death observed was due to necroptosis by performing a western blot analysis of phosphorylated MLKL. At 12 h post-infection, western blot analysis reveals that MYXV infection results in the accumulation of phosphorylated MLKL. However, inhibition of RIPK1 and RIPK3 with GSK’963 and GSK’872 prevented the accumulation of pMLKL (Fig. 10C). These results indicate that MYXV-mediated activation of necroptosis is functional in both human and mouse cells and depends on the canonical components of the necroptosis machinery.

### Activation of necroptosis inhibits MYXV replication in mouse L929 cells

**Fig 11:**
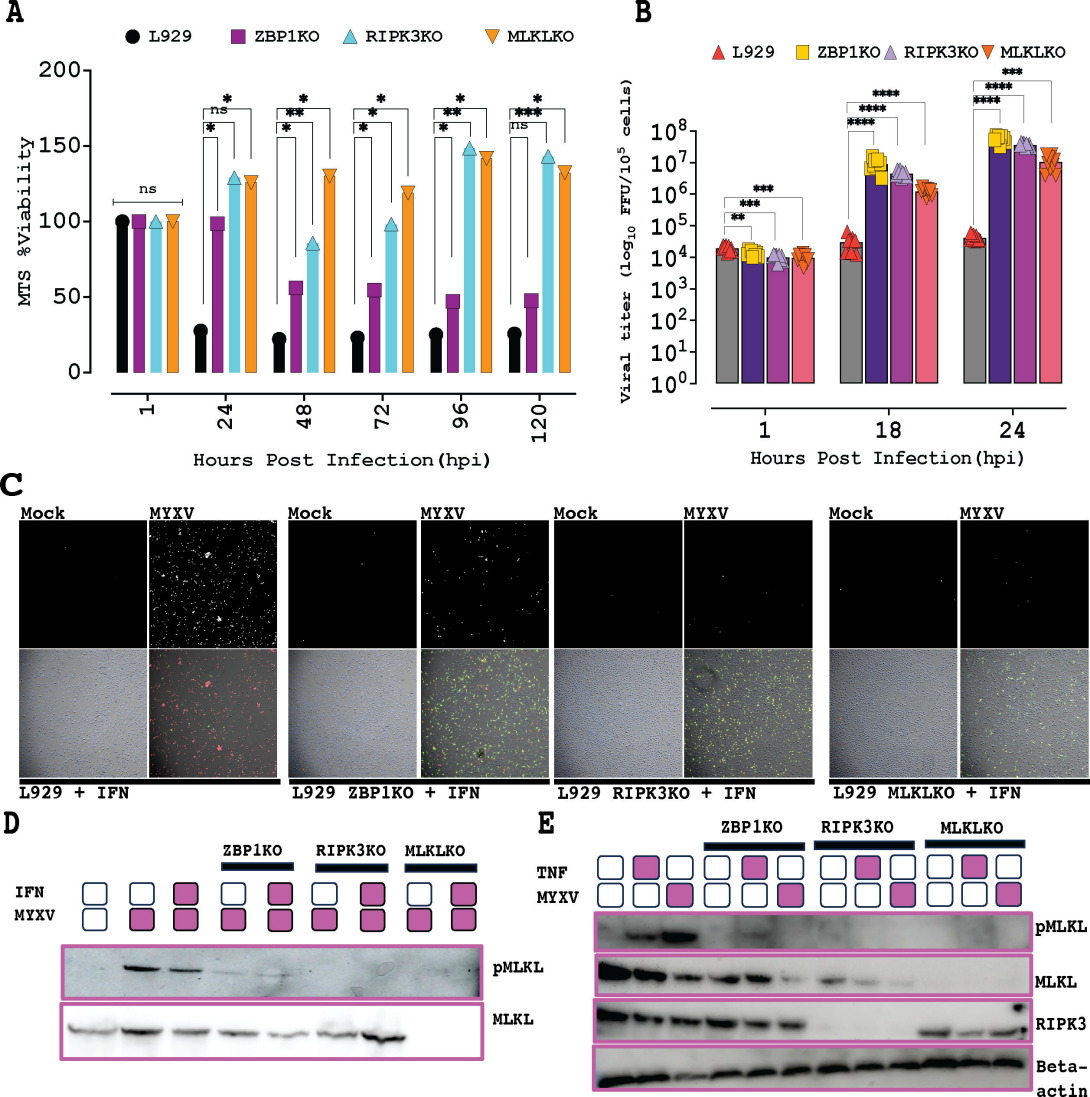
Necroptosis inhibits MYXV replication in mouse L929 cells. (**A**) MTS assay measuring cell viability at 1, 24, 48, 96, and 120 h after virus infection in naïve L929, L929-ZBP1KO, L929-RIPK3KO, and L929-MLKLKO cells. (**B**) MYXV titration in naïve L929, L929-ZBP1KO, L929-RIPK3KO, and L929-MLKLKO cells at 1, 18, and 24 h.p.i. (**C**) Fluorescence images of IFN-primed naïve L929, L929-ZBP1KO, L929-RIPK3KO, and L929-MLKLKO cells, either left uninfected (mock) or infected with MYXV expressing GFP. (**D**) Western blot probing pMLKL and MLKL in the presence and absence of IFN and MYXV, in the cell lines mentioned in Fig. 3, harvested at 24 h after infection. (**E**) Western blot for pMLKL, MLKL, RIPK3, and beta-actin for TNF-primed sample treatments as in panel D, in the presence of MYXV, TNF-alpha, or no treatment.

To further understand the effects of various necroptotic components on MYXV replication, we used L929 cells genetically modified to lack ZBP1, RIPK3, or MLKL. First, we tested the effect of MYXV on the viability of these cell lines by MTS assay. At 24 hpi, the viability of unmodified naive L929 cells dropped to about 25% (Fig. 11A), and cell viability remained at 25% for the next several days tested (Fig. 11A). ZBP1 knockout cells exhibited almost 100% viability at 24 hpi compared to naive L929 cells (Fig. 11A). However, at 48 hpi, ZBP1-KO L929 cell viability dropped to about 55% and remained at that level for several days. Surprisingly, RIPK3 and MLKL knockout L929 cells had minimal or no effect on cell viability after MYXV infection over the 5-day course of the experiment (Fig. 11A). Next, we quantified the impact of necroptotic components on virus replication. ZBP1, RIPK3, and MLKL knockout in L929 cells increased MYXV titers–a 3-log increase on average. Compared to the L929 knockout cell lines, naive L929 cells showed little to no increase in viral titers (Fig. 11B). Since the deletion of ZBP1, RIPK3, and MLKL is expected to affect necroptosis, we compared the effects of MYXV infection on naive L929 cells and on L929 cells lacking either ZBP1, RIPK3, or MLKL. Cells were seeded in the presence or absence of IFN. IFN treatment progressed for 24 h, and the cells were infected with vMyx-GFP (wild-type MYXV construct expressing enhanced green fluorescence protein [gfp] under a poxvirus synthetic early/late promoter) (Fig. 11C). At 24 h post-infection, the cells were harvested and subjected to western blotting analysis. RIPK3 and MLKL knockouts had no MLKL phosphorylation on the blots (Fig. 11D and E). We detected the phosphorylation of mouse MLKL in naive L929 cells in the presence and absence of IFN (Fig. 11D). ZBP1 knockout cells had some trace amount of phosphorylated MLKL in the presence and absence of IFN, although not comparable to naive L929 cells, suggesting that ZBP1 might not be the only pathway that leads to MYXV-dependent necroptosis in mouse L929 cells (Fig. 11D). We repeated the same experiment as before in the presence of TNF, and samples were collected 8 h post-infection and subjected to western blot analysis. We detected MLKL phosphorylation in naive L929 cells treated with either MYXV or TNF, but not in ZBP1, RIPK3, and MLKL knockout cells (Fig. 11E). Unexpectedly, we observed that ZBP1 deletion affected TNF-dependent necroptosis in mouse L929 cells, suggesting that ZBP1 might play an integral role in the signaling of TNF-dependent necroptosis. Thus, Sytox staining confirmed that gene knockouts of ZBP1, RIPK3, and MLKL rescued the viability and replication of MYXV virus-infected cells (Fig. 11C). Together, our data suggest that necroptosis inhibits MYXV replication in necroptosis-competent non-permissive cells. This confirms that MYXV does not contain a functional inhibitor of necroptosis.

### Effects of extracellular TNF and VACV E3 protein on MYXV-mediated necroptosis

**Fig 12:**
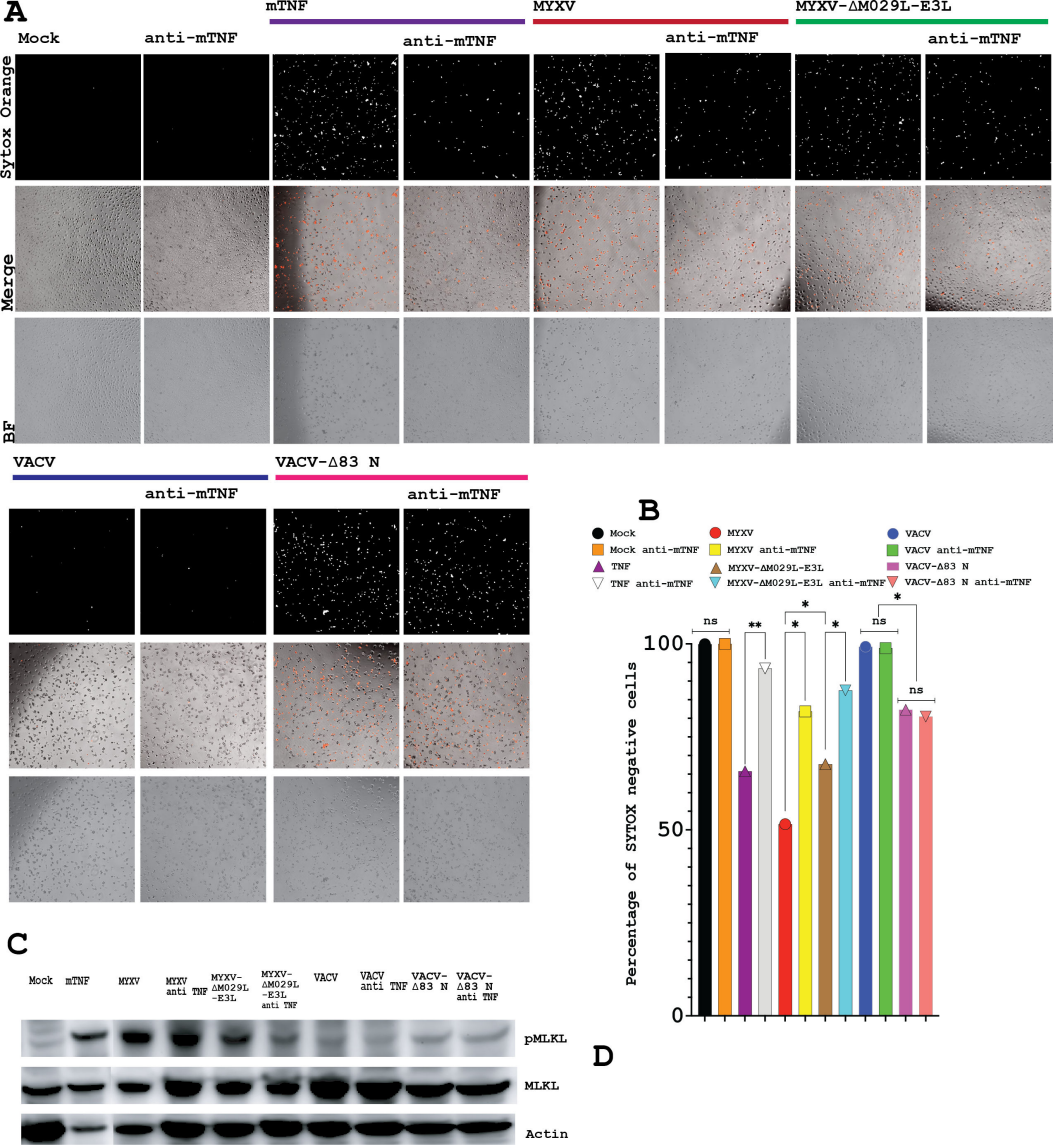
Extracellular TNF inhibition and expression of VACV E3 rescues MYXV-mediated necroptosis cell death in mouse cells. (**A**) Fluorescence images of IFN-primed L929 cells with the following treatments: Untreated (Mock), Mock + mTNF, Mock + anti-mTNF, Mock + TNF, Mock + TNF + Anti-TNF, MYXV, MYXV + Anti-TNF, vMyx-ΔM029-E3L, vMyx-ΔM029-E3L + Anti-TNF. At 24 h post-treatment, cells were treated with Sytox orange dye, and images were captured. (**B**) Cell viability was calculated by counting SYTOX-positive cells relative to the total number of cells. (**C**) Western blot measuring the presence of pMLKL, MLKL, and beta-actin. Sample treatments were as described for panel A.

To investigate the extent to which intracellular and extracellular necroptosis are affected during MYXV infection, we constructed MYXV expressing the VACV E3 protein in place of M029 (vMyx-ΔM029L-E3L). The newly constructed virus has shown no defect in viral replication (data not shown). We hypothesize that vMyx-ΔM029L-E3L should partially restore cell viability during necroptosis, as MYXV activates both RIPK1- and RIPK3-dependent pathways. To test the effect of vMyx-ΔM029L-E3L, mouse L929 cells were seeded in the presence of IFN. After 24 h, the cells were either left untreated or treated with 1 µg/mL of mouse TNF antibody for 30 min, followed by treatment with the following necroptosis-related agonists: TNF, MYXV, vMyx-M029L-E3L, VACV, and VACV-Δ83N. The infection proceeded for 24 h, and the cells were treated with Sytox orange dye and imaged using a fluorescent microscope. Red-fluorescence objects were thresholded and counted and used as a measure of dead cells. We observed that, under agonist-treated conditions, mouse TNF antibody treatment rescued TNF, MYXV, and vMyx-ΔM029L-E3L treatments, but not VACV or VACV-Δ83N, consistent with previous findings (4), and highlighting the role of extrinsic necroptosis in regulating MYXV necroptosis. Importantly, vMyx-ΔM029L-E3L increased the number of necroptosis-negative cells compared to the wild-type MYXV, suggesting a possible protective role of the E3 protein during MYXV infection in necroptosis-competent cells (Fig. 12A and B). Western blot analysis revealed that both vMyx-ΔM029L-E3L and MYXV accumulated phosphorylated MLKL, as did the TNF-treated cells (Fig. 12C). In summary, our results show that MYXV expressing the E3 protein modestly increases cell viability and that secreted TNF-α drives MYXV-mediated necroptosis.

## DISCUSSION

In this study, we defined the diversity of dsRNA-BD fold-containing proteins within poxviruses. Our data support the notion that poxviruses contain three unique categories of dsRNA-BD fold-containing proteins of independent origin. Structural comparison of the identified dsRNA-BD fold-containing proteins with those of VACV E3 revealed significant similarity within all three categories of dsRNA-BD fold-containing proteins, suggesting that these proteins are core immune modulators of poxvirus infection. Traditionally, based on protein sequence homology, it was known that molluscipoxviruses lacked an E3 orthologous protein that regulates PKR, possibly due to high sequence divergence between the MC141 protein and E3-like proteins (7, 31). Our structural homology comparison, in combination with the identification of key dsRNA-BD residues in macropoxvirus and molluscipoxvirus, for the first time shows that these viruses contain dsRNA-BD-containing proteins. (Fig. 1A). Although dsRNA binding is the major hallmark of dsRNA domain-containing proteins, it is also possible that the newly identified dsRNA-BD-fold-containing proteins in macropoxvirus and molluscipoxvirus perform functions unrelated to dsRNA binding, since all the identified proteins lack additional residues critical for dsRNA binding present in VACV E3 (28, 32). It is possible that non-canonical dsRNA-fold-containing proteins are involved in host-specific protein-protein interactions. Additional experiments would be needed to validate whether the newly identified proteins can bind dsRNA, regulate canonical pathways such as PKR, and engage in other protein-protein interactions.

We previously reported that specific mammalian lineages are missing key proteins in the necroptosis pathway, and their accompanying poxviruses lack the N-terminus Z-nucleic acid-binding domain, which is critical for necroptosis inhibition (3). In this work, we identified members from four different families of poxviruses that are fully or partially missing the N-terminus Zα-BD: leporipoxvirus (MYXV-Lau, MYXV-Tol, and SFV), waddensea poxvirus, cetacean poxvirus, and orthopoxvirus (Mpox and volepox) (Fig. 5A). Recurrent Zα-BD loss amongst different poxvirus families seems to be an independent event (Fig. 3A and 5A). Unexpectedly, we observed that the N-terminus domain of the E3-like homologous proteins appears to bear additional secondary structure when compared to the canonical Zα-BD in VACV, suggesting a divergence in protein function (Fig. 5D and E). However, this was not the case for the dsRNA-BD (Fig. 5D and E). These observations suggest that the dsRNA-BD might be under higher selective pressure than the Zα-BD. However, we do not yet have a good theoretical explanation for the recurrent loss and extensive modifications at the N-terminus domain of E3.

The structures of the E3-like proteins missing their Zα domain have a FATCAT score greater than 238 when compared with the dsRNA-BD of VACV (Fig. 5A through E). This suggests conservation in the dsRNA-BD within the poxvirus family. On the other hand, the N-terminal modification seems to be unique for each poxvirus, suggesting a varying degree of adaptation at the N-terminus domain. In all, poxviruses lacking an N-terminus Zα-BD contain unique secondary structures at the N-terminus that might have adapted for diverse host-specific functions from Zα-nucleic acid regulation.

In this work, we compared selected members of orthopoxviruses, known to inhibit necroptosis, with leporipoxvirus, which we hypothesized would activate the necroptosis pathway because their host species of origin lacks key regulators of necroptosis (3, 4, 20, 33). In other words, leporipoxviruses contain no functional inhibitors of necroptosis because there is no selection pressure to do so. Our data suggest that in human necroptosis-competent cells, leporipoxviruses promoted cell death and the accumulation of pMLKL to levels higher than those observed with wild-type orthopoxviruses. This indicates that leporipoxviruses lack counter-adaptation to necroptosis or do not encode any functional inhibitors of the pathway (Fig. 7). Although leporipoxviruses stimulated necroptosis to levels greater than wild-type orthopoxviruses, VACV, missing the Zα-BD (VACV-Δ83N), promoted rapid cell death and a more significant amount of pMLKL than any of the leporipoxviruses. Thus, leporipoxviruses did not phenocopy VACV-Δ83N in terms of necroptosis triggering. We think the higher cell death observed with VACV-Δ83N is due to orthopoxviruses replicating at faster kinetics than leporipoxviruses in human and mouse-derived cells, based on the assumption that viral nucleic acid accumulation reflects the level of active virus replication.

In both human and mouse-derived necroptosis-competent cells, we observed that RIPK1, RIPK3, and MLKL all contribute to the MYXV-induced necroptosis to varying degrees. Treatment with both RIPK1 and RIPK3 inhibitors partially rescued MYXV-induced necroptotic cell death (Fig. 9 and 10). However, the MLKL inhibitor NSA almost entirely rescued cell death (Fig. 9). These observations support the involvement of RIPK1 and RIPK3 in the MYXV-induced activation of necroptosis. Koehler et al. previously demonstrated that the VACV lacking the Zα-NA binding domain activates RIPK3, not RIPK1 necroptosis (4). These differences between MYXV and VACV suggest that other inhibitors of the necroptosis pathway may be present in VACV but absent in MYXV. Apart from poxviruses, many viruses target RIPK1, RIPK3, and MLKL to inhibit necroptosis (34). For example, herpesvirus-encoded RHIM proteins M45, ICP6, and ICP10 impede the formation of RIPK3 amyloid, and Epstein-Barr virus (EBV)-encoded latent membrane protein 1 (LMP1) blocks the formation of RIPK1 and RIPK3 necrosome (35).

Furthermore, we explore the extent to which the N-terminus of E3 affects necroptosis and cell death by using a MYXV construct expressing VACV E3 protein (Fig. 12). E3-expressing MYXV exhibited higher cell viability compared to wild-type MYXV, suggesting a potential role of the Zα-NA-binding domain of E3 in cell death regulation.

Using MYXV, we demonstrate that viruses lacking functional inhibitors of necroptosis can serve as a model to understand the roles of individual components of the necroptosis pathway in activating cell death. Additionally, our studies demonstrate that the functional necroptosis pathway inhibits MYXV replication and the formation of infectious progeny virions. This is possibly one of the mechanisms by which MYXV is restricted in hosts other than leporids. These results raise the question of why rabbits lost the necroptosis pathway. Additionally, it raises the question of how a myxomatosis disease state would be affected by the presence of a functional necroptotic system.

Necroptosis is an inflammatory form of cell death that has been shown to have varying effects on cancer progression, metastasis, and clearance (33, 36–38). On the other hand, MYXV is currently being developed as an oncolytic virus for cancer treatment (39, 40). Understanding the effects of MYXV-induced necroptosis, a potential form of immunogenic cell death, on cancer therapy or on the efficacy of MYXV as an oncolytic virus could improve our understanding of cancer immunotherapy.

## MATERIALS AND METHODS

### National Center for Biotechnology Information (NCBI) BLAST

Vaccinia virus E3 protein sequence (AGJ91218.1) was queried against the poxvirus database on NCBI using the DELTA BLAST function. We repeated the same BLAST run using crocodile poxvirus (YP_784347.1 CRV157) as the query against poxvirus families that were not identified in the initial screen (molluscipoxvirus, macropoxvirus, avipoxvirus, and entomopoxvirus). All the BLAST processes were run with the default settings on the NCBI.

### AlphaFold singularity and protein structure visualization

The E3-Like FASTA files for all poxviruses used in our screen were obtained from NCBI. FASTA files were submitted to the AlphaFold2 singularity at the Arizona State University Research Computing Core. Computer-generated Protein Data Bank (PDB) files were visualized and overlaid using UCSF ChimeraX Daily, developed by the Resource for Biocomputing, Visualization, and Informatics at the University of California, San Francisco, with support from NIH P41-GM103311 (41).

Python scripts were implemented to run TM-Align and FATCAT on our local supercomputing cluster (24, 25). RAW data were graphed in GraphPad Prism.

### Phylogenetic analysis

The poxvirus phylogenetic tree was constructed using the DNA polymerase enzyme, homologs to the vaccinia virus E9 protein. The polymerase amino acid sequence files were aligned using MUSCLE as implemented by MEGA 11. The E3L-like FASTA files from our initial NCBI search were aligned using MUSCLE as implemented in MEGA 11 (42). All the aligned FASTA files on our screen were submitted to IQ-Tree 2 using the default settings to build a phylogenetic tree (43). FigTree was used to visualize tree files obtained from IQ-Tree (43). FigTree files were visualized and illustrated using Adobe Illustrator. For the structural comparison involving the dsRNA-BD and Zα-BD domains of E3 proteins, only viruses that contained complete Zα-BD domains were used in our comparison.

### Synteny analysis

Genomes (GenBank format) for representative poxviruses from each family were downloaded from the NCBI. The files were trimmed to allow 10 genes on both sides of the dsRNA-BD-containing protein. Synteny analysis was performed using the default setting on clinker & clustermap.js (44).

### RNA sequencing

Human HT29 cells were grown in 100 mm cell culture dishes. Samples were either treated with IFN or left untreated. Cells were harvested 24 h post-treatment and subjected to RNA isolation. RNA was sent to Novogene for RNA sequencing. Sequencing outputs were subjected to a standard data analysis pipeline that included differential gene expression and functional enrichment analysis using R. Gene count matrix file was used to create a heatmap of the genes involved in the establishment of necroptosis.

### Cells

Mouse L929, L929-ZBP1-KO, L929-RIPK3-KO, L929-MLKL-KO, JC, and JC-PKR-KO were obtained from Dr. Jacobs Bertram’s lab at ASU. HT29 (catalog no. HTB-38), RK13 (catalog no. CCL-37), and Colo205 (catalog no. CCL-222) were purchased from the American Type Culture Collection (ATCC). All the cell lines were tested for mycoplasma contamination before experimentation using the Universal Mycoplasma Detection Kit from ATCC (ATCC# 30-1012K). L929s were maintained in Minimum Essential Medium (MEM) (CORNING # 10-022-CV) supplemented with 5% FBS and/or 100 µg penicillin-streptomycin (P/S; Cytiva #SV30010). HT29 was maintained in McCoy’s (Cytiva #SH30200.FS), 10% FBS, and 100 µg P/S. Colo205 and JC cells were cultured in RPMI (Roswell Park Memorial Institute) (Cytiva #SG30027.02) with 10% FBS and 100 µg P/S. Rabbit RK13 cells were maintained in Dulbecco’s modified Eagle’s medium (Cytiva #SG30022.LS) supplemented with 10% FBS and 100 µg P/S. All cells were maintained at 37 °C in a 5% humidified incubator.

### Viruses

For all necroptosis experiments, human and mouse cell lines were infected at an MOI of 5. Wild-type MYXV-Lausanne (MYXV-Lau), vMyx-GFP (wild-type MYXV that expresses GFP under a poxvirus synthetic early-late promoter), vMyx-GFP-tdTomato (wild-type MYXV that expresses GFP under a poxvirus synthetic early-late promoter and tdTomato under a poxvirus p11 late promoter), vMyx-Tol (wild-type MYXV-Toledo that expresses GFP under a poxvirus synthetic early-late promoter and tdTomato under a poxvirus p11 late promoter) virus constructs, as described before, were used in different experiments (45–47). The SFV virus construct expressing the reporter lacZ gene was described before (48). vMyx-ΔM029L-E3L virus was generated by homologous recombination using a DNA fragment containing E3L under the natural M029 promoter with flanking sequences from M028L and M030L, using the methods described previously (5).

We used the VACV Western Reserve strain, expressing GFP under a poxvirus synthetic early-late promoter and tdTomato under a poxvirus p11 late promoter (45). CPXV Brighton Red strain expressing GFP under a poxvirus synthetic early-late promoter was reported previously by Lui et al. (20). These viral constructs were used, as described before (46). Myxoma virus stocks were obtained through sucrose gradient purification (49). Wild-type VACV and VACV-83N Western Reserve strain was obtained from Dr. Jacobs Bertram’s lab at Arizona State University and described previously (4).

### Virus titration

Cells were seeded in 24-well plates a day before infection and, on the day of the experiment, infected at the appropriate MOI. The virus was allowed to adsorb for 1 h, then washed off with phosphate-buffered saline (PBS) and replaced with fresh media. Cell pellets and supernatants were harvested at different time points after infection and frozen at −80℃ until processed. Samples were subjected to three freeze-thaw cycles and sonicated to release cell-associated virions. Samples were titrated in RK13 cells (49).

### Antibodies

Human pMLKL (Abcam #187091, Cell Signaling #91689S), human MLKL (Abcam #ab243142, ab172868, ab184718, Cell Signaling #14993S), human RIPK1 (Cell Signaling #3493S), human phospho-RIPK1 (Cell Signaling #65746S), human RIPK3 (Cell Signaling #13526), human phospho-RIPK3 (Cell Signaling #57220S), human ZBP1 (Novus Biologicals #NBP1-76854), beta-actin (Life Technologies #MA5-15739-HRP), mouse pMLKL (Abcam #ab196436), mouse MLKL (Cell Signaling #37705S), mouse RIPK3 (Cell Signaling #95702), mouse TNF-alpha (R&D Systems #AF-410-NA, Invitrogen #16-7321-85), J2 dsRNA (Nordic-MUbio #SCICONS J2 – 10010200), and Z-NA (Novus Biologicals #NB100-749).

### Western blotting and phospho-protein detection

For western blotting, protein samples were harvested, pelleted, and lysed using Radioimmunoprecipitation assay buffer (RIPA) in the presence of a protease-phosphatase inhibitors cocktail (Thermo Fisher Scientific #78446). Laemmli buffer (BIO-RAD #1610747) or SDS buffer containing 5% beta-mercaptoethanol was added to samples and heated at 95°C for 10 min, and a Sodium Dodecyl Sulfate (SDS) protein gel was run. The SDS gel was transferred to a membrane and blocked with 1% bovine serum albumin (BSA) in Tris-buffered saline with 0.1% Tween (TBST) buffer for 1 h. Next, the blot was incubated overnight with the primary antibody, washed with TBST for 5 min three times, and incubated with a secondary antibody conjugated to HRP. The membrane was washed with TBST to get rid of the secondary antibody. Membranes were visualized using an Amersham ImageQuant (Cytiva) 800 western blot imager.

### Cell treatment and Sytox cell viability assay

Cells were seeded the day before the experiment and treated with media containing and/or 100 µg mL^−1^ Benzyloxycarbonyl-Val-Ala-Asp(OMe)-fluoromethylketone (Z-VAD-FMK) (ApexBio #1902), 2 µg mL^−1^ 1-[(5S)−4,5-Dihydro-5-phenyl-1H-pyrazol-1-yl]−2 (GSK’963) (Sigma-Aldrich #SLM2376-25MG), 2 µg mL^−1^ N-(6-(isopropylsulfonyl)quinolin-4-yl)benzo[d]thiazol-5-amine hydrochloride (GSK’872) (Sigma-Aldrich #5303890001), 2 µg mL^−1^ Necrosulfanamide (EMD Millipore #480073), and 1 µg mL^−1^ of Sytox Orange (Invitrogen #S11368). Samples were observed using the EVOS live cell imaging system, and representative images from different time points were quantified by counting the dead cells that contained the Sytox dye compared to the live cells that incorporated the nuclei stain Hoechst (Thermo Fisher Scientific #62249).

### MTS cell viability assay

Cells were seeded in a 96-well plate to a 90% confluency and infected with vMyx-GFP-tdTomato. At the appropriate time points, the MTS solution (3-(4,5-dimethylthiazol-2-yl)−5-(3-carboxymethoxyphenyl)−2-(4-sulfophenyl)−2H-tetrazolium) (Promega #G3581) was added to individual wells. Samples containing MTS solution were incubated for 1 h, and the absorbance at 490 nm was recorded using a microplate reader (Thermo Scientific, Varioskan LUX multimode microplate reader). Time points were recorded in triplicate.

### Immunofluorescence assay and fluorescence imaging

Cells were seeded overnight on an eight-well Ibidi dish and infected with different viruses. At the appropriate time points, samples were fixed using paraformaldehyde (PFA) for 5 min, rinsed with PBS, and permeabilized using Triton X-100 for 2 min. After permeabilization, the sample was fixed with PFA for 5 min and blocked in 1% BSA for 1 h at room temperature. The samples were incubated with the primary antibody overnight and washed with PBS supplemented with 0.1% Tween (PBST). The secondary antibody conjugated to the fluorophore (Thermo Fisher Scientific, Alex Fluor 488, 568, and 647) was incubated for 1 h at room temperature. The secondary antibody was washed off using PBST, and samples were immersed in PBS containing 1 μg/mL of 4′,6-diamidino-2-phenylindole (DAPI) and imaged using a Nikon AX confocal microscope at the Biodesign Imaging Core Facility. Samples with varying treatments were imaged using a Nikon Eclipse Ti2 microscope (Nikon). The images were converted using Nikon software.

### Data analysis and figure design

All experiments were performed at least three times independently. A two-way ANOVA and an unpaired *t*-test at a 95% confidence level, as implemented in GraphPad Prism, were used to compare treatments. *P* values are reported as follows: not significant (ns), *P* > 0.05; *, *P* < 0.05; **, *P* < 0.01; ***, *P* < 0.001; ****, *P* < 0.0001. All figures were designed using Adobe Illustrator, except for the graphical abstract, which was created using BioRender.

## Data Availability

All data supporting the findings of this study are available within the article. RNA-seq data have been deposited in the NCBI database under accession number PRJNA1467770. Accession numbers for the poxviruses used in this study are as follows: squirrelpox virus (HE601899), pteropox virus (KU980965), parapox virus (KM502564), bovine papular stomatitis virus (AY386265), eptesipox virus (KY747497), cetacean poxvirus (MN653921), yokapox virus (HQ849551), vaccinia virus Western Reserve (AY243312), Yaba monkey tumor virus (AY386371), lumpy skin disease virus (ON152411), cotia virus (HQ647181), myxoma virus (AF170726), Western grey kangaroopox virus (MF467280), molluscum contagiosum virus (BK012038), Wadden seapox virus (OP810554), volepox virus (KU749311), Nile crocodilepox virus (DQ356948), saltwater crocodilepox virus (MK903863), monkeypox virus Zaire (AF380138), cowpox virus Brighton Red (AF482758). Any additional information is available from the authors upon request.

## SUPPLEMENTAL MATERIAL

Fig. S1 jvi.00114-26-s0001.tiff AlphaFold structures of dsRNA fold-containing proteins of selected poxviruses merged with VACV E3.

Fig. S2 jvi.00114-26-s0002.tiff Amino acid sequence alignment of poxvirus-encoded dsRNA fold-containing proteins.

Supplemental legends jvi.00114-26-s0003.docx Descriptive legends for Fig. S1 and S2.

Graphical abstract jvi.00114-26-s0004.tiff Visual depiction of the study.

ASM does not own the copyrights to Supplemental Material that may be linked to, or accessed through, an article. The authors have granted ASM a non-exclusive, world-wide license to publish the Supplemental Material files. Please contact the corresponding author directly for reuse.
