# Supplementary figures and images for: Divergence in poxvirus-encoded E3-like proteins can dictate poxvirus activation of cellular necroptosis
Source: J Virol. 2026 Jun 17;100(7):e00114-26. doi: 10.1128/jvi.00114-26 (PMC13386979; doi:10.1128/jvi.00114-26)

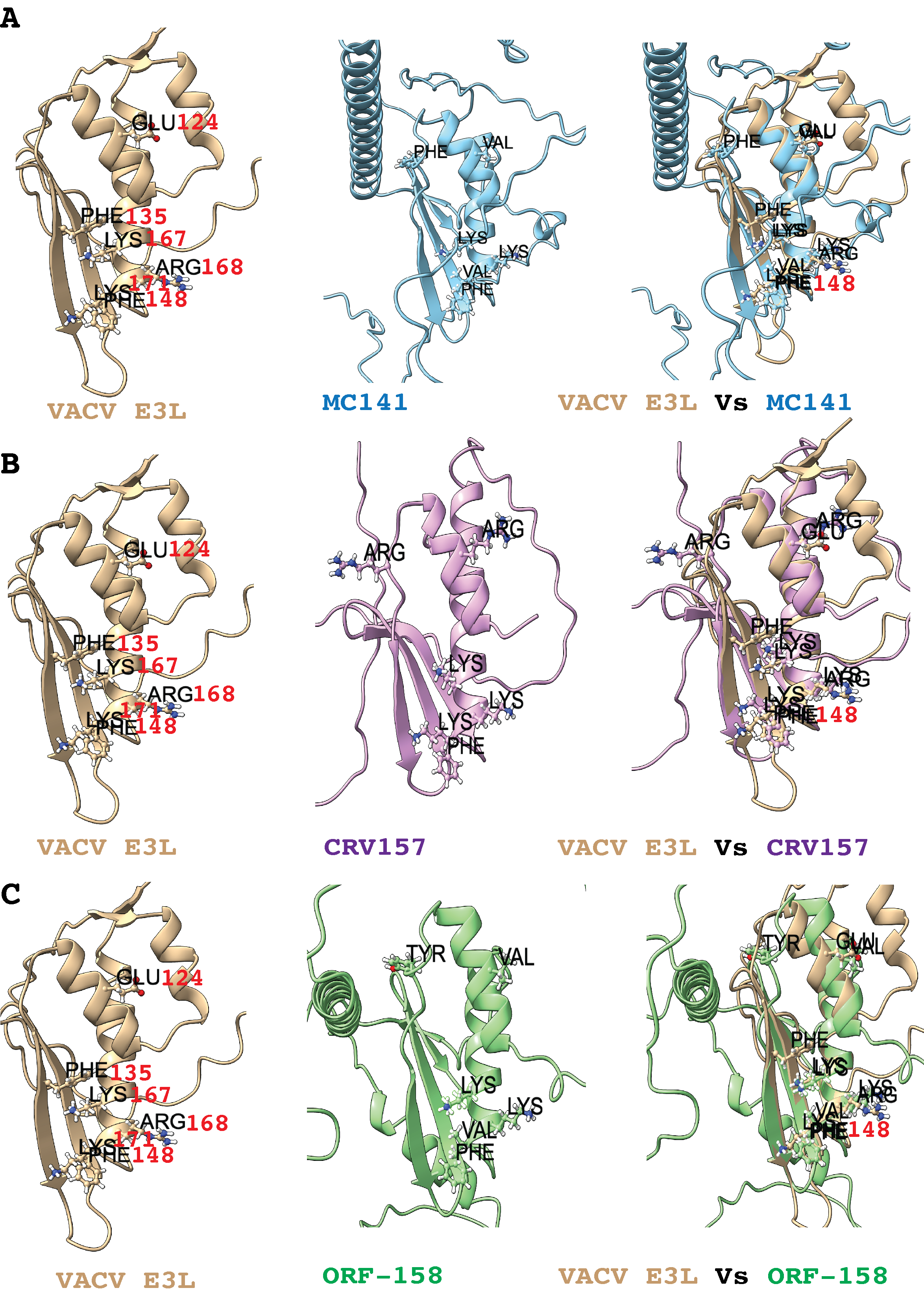

Supplement: Fig. S1 — AlphaFold structures of dsRNA fold-containing proteins of selected poxviruses merged with VACV E3. [file jvi.00114-26-s0001.tiff]

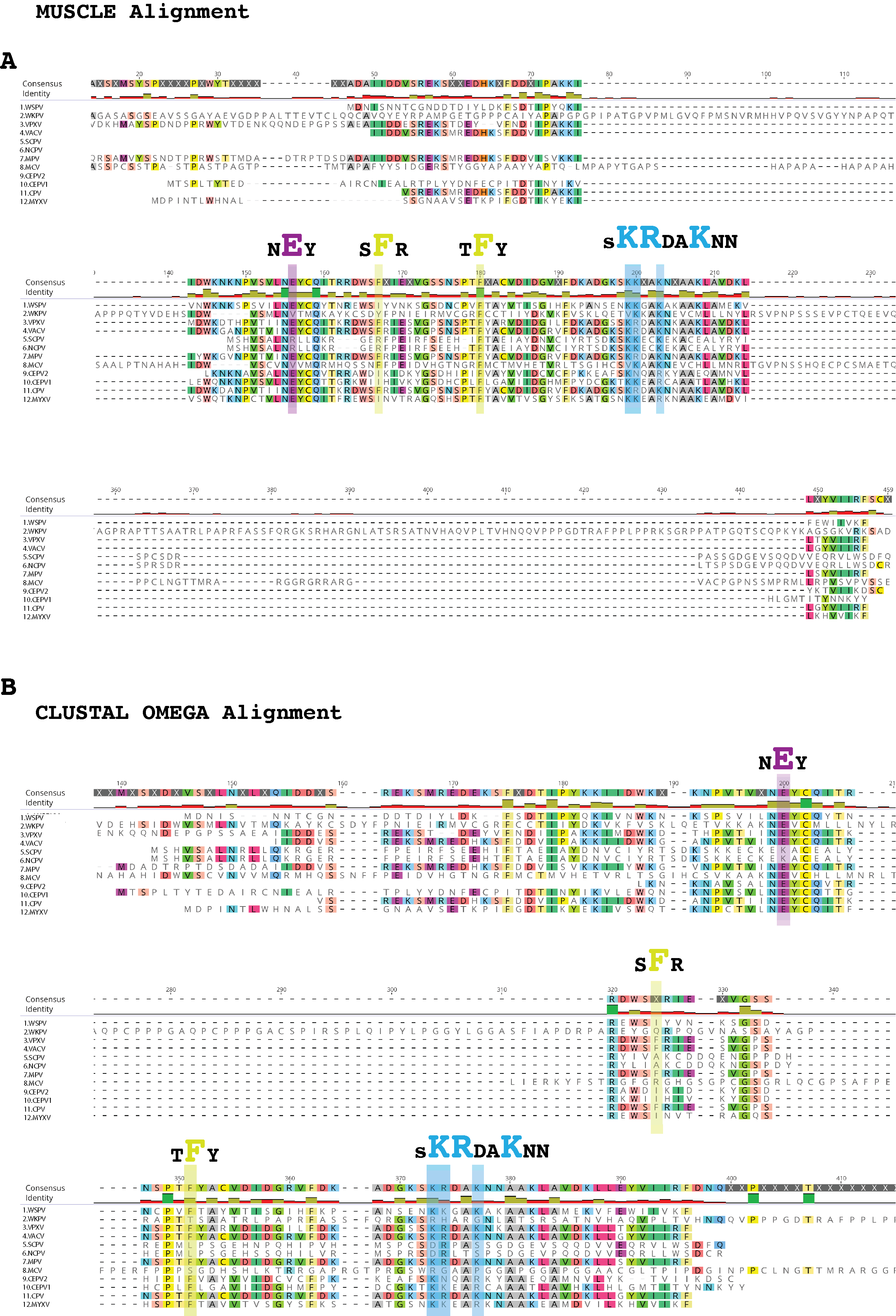

Supplement: Fig. S2 — Amino acid sequence alignment of poxvirus-encoded dsRNA fold-containing proteins. [file jvi.00114-26-s0002.tiff]

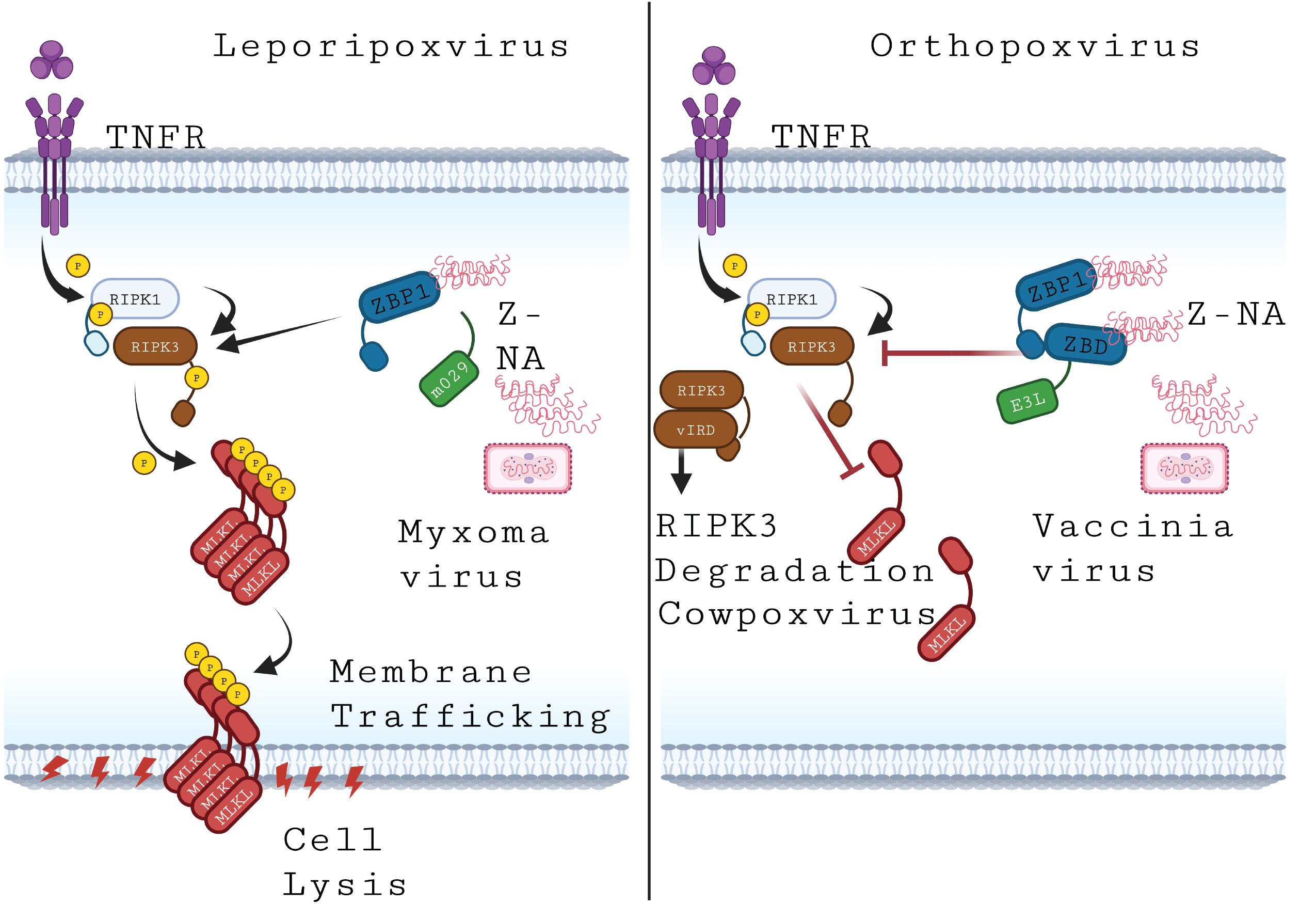

Supplement: Graphical abstract — Visual depiction of the study. [file jvi.00114-26-s0004.tiff]
