# Supplementary material for: Divergence in poxvirus-encoded E3-like proteins can dictate poxvirus activation of cellular necroptosis
Source: J Virol. 2026 Jun 17;100(7):e00114-26. doi: 10.1128/jvi.00114-26 (PMC13386979; doi:10.1128/jvi.00114-26)
Supplement: Supplemental legends — Descriptive legends for Fig. S1 and S2. [file jvi.00114-26-s0003.docx]

Supplementary file S1:

Alpha fold structure of dsRNA-fold containing proteins of VACV E3, either alone or merged with (A) molluscum contagiosum MC141, (B) Nile crocodile poxvirus CRV157, and (C) Western grey kangaroo poxvirus ORF-158. Three-letter symbols of amino acids involved in dsRNA binding in VACV E3 are highlighted in black.

Supplementary file S2:

Amino acid sequence alignment of poxvirus-encoded dsRNA fold-containing proteins from select members of the 3 major classes of proteins identified (A) aligned using MUSCLE and (B) CLUSTAL OMEGA. Amino acids involved in dsRNA binding in VACV E3 are highlighted in pink, yellow, and blue.
